# Targeting PD‐L1 with DNA Aptamers and Conjugated with Gemcitabine as a Novel Therapeutic Strategy for Bladder Cancer Chemotherapy Combined with Immunotherapy

**DOI:** 10.1002/smsc.202300104

**Published:** 2023-10-17

**Authors:** Xing Hu, Hongliang Zeng, Yongbo Peng, Minhua Deng, Wei Xiang, Biao Liu, Jiahao Liu, Yunlong Fu, Zhiqiang Hu, Weibin Hou, Xuewen Liu, Jin Tang, Zhi Long, Long Wang, Jianye Liu

**Affiliations:** ^1^ Department of Urology The Third Xiangya Hospital of Central South University No.138, Tongzipo Road Changsha Hunan 410013 China; ^2^ Center of Medical Laboratory Animal Hunan Academy of Chinese Medicine Changsha Hunan 410013 China; ^3^ Chongqing Key Laboratory for Pharmaceutical Metabolism Research College of Pharmacy Chongqing Medical University No.1, Yixueyuan Road Chongqing 400016 China; ^4^ Department of Urology Sun Yat-sen University Cancer Center No. 651, Dongfeng Road East Guangzhou Guangdong 510060 China; ^5^ State Key Laboratory of Oncology in Southern China Collaborative Innovation Center for Cancer Medicine No. 651, Dongfeng Road East Guangzhou Guangdong 510060 China; ^6^ Department of Oncology The Third Xiangya Hospital of Central South University No.138, Tongzipo Road Changsha Hunan 410013 China

**Keywords:** bladder cancer, chemotherapy, immunotherapy, PD-L1 aptamers, targeted therapy

## Abstract

Due to the poor stability and adverse effects of chemotherapy drugs, such as gemcitabine, the current effectiveness of traditional chemotherapy is minimal. Some patients also show a low response rate to immunotherapy. Therefore, a novel material PD‐L1‐GEMs is designed and synthesized with targeted specificity. PD‐L1‐GEMs specifically bind to bladder cancer cells. Free gemcitabine cleaved by a phosphatase enters bladder cancer cells through the macropinocytosis pathway and induces cytotoxicity. PD‐L1‐GEMs show good stability, binding specificity, and significant inhibitory effects in vitro. Two bladder tumor models (subcutaneous model and in situ model) show inhibition of growth and progression in PD‐L1‐GEMs treatment, as well as good biosafety in vivo. The PD‐L1 aptamer blocks the binding of PD‐L1 on the tumor cell surface to PD‐1 on T lymphocytes, restoring their immune function, inducing cytokine production and aggregation, and exerting an immune killing role on bladder cancer cells. PD‐L1‐GEMs represent a successful chemotherapy–immunotherapy strategy for bladder cancer.

## Graphical abstract

PD‐L1‐GEMs are generated by coupling PD‐L1 aptamer and gemcitabine through aptamer–drug conjugates. The materials deliver gemcitabine accurately and efficiently through targeting ability and play a role in chemotherapy and immunotherapy for bladder cancer.

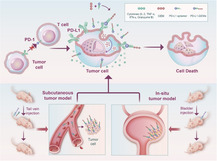

## 1 Introduction

Bladder cancer is one of the most common malignant tumors in the urinary system, with the highest mortality rate. Clinically, it is mainly divided into muscle‐invasive bladder cancer (MIBC) and nonmuscle invasive bladder cancer (NMIBC).^[^ ^1^ ^]^ Surgical treatment and chemotherapy have been the most commonly used treatments for bladder cancer. Although improvements in surgical treatment methods and combinations of chemotherapy agents (GC scheme: gemcitabine and cisplatin) have worked well in patients with bladder cancer,^[^ ^2^ ^]^ the majority of patients will still face many problems, such as recurrence or tumor metastasis, chemotherapy drug resistance, and serious side effects.^[^ ^3^ ^]^ To treat bladder cancer, the main first‐line chemotherapeutic agent is gemcitabine (GEM), which interferes with cell proliferation by inhibiting cellular DNA synthesis, leading to tumor cell apoptosis.^[^ ^4^ ^]^ GEM is strongly soluble, but shows weak tumor affinity, frequently resulting in poor clinical efficacy and serious adverse events.^[^ ^5^, ^6^ ^]^ Thus, there is an urgent need to find a new treatment strategy that can enhance the therapeutic effect of chemotherapy for bladder cancer while reducing toxic side effects.

To improve bladder cancer treatment, several other therapeutic methods have emerged, such as immunotherapy, targeted therapy, and gene therapy.^[^ ^7^, ^8^, ^9^ ^]^ Currently, immunotherapy is mainly represented by immune checkpoint inhibitors (ICIs),^[^ ^10^ ^]^ especially programmed cell death 1 (PD‐1)/programmed cell death ligand 1 (PD‐L1) checkpoint inhibitors,^[^ ^11^, ^12^, ^13^ ^]^ which exert killing effects on tumor cells by inhibiting the immune escape of tumor cells and restoring the normal function of immune cells. The use of ICIs is a major breakthrough in tumor treatment. However, only about 30% of patients respond to immunotherapy^[^ ^14^, ^15^ ^]^; therefore, the range of patients who benefit is small. Moreover, a study reported that neoadjuvant therapy combined with chemotherapy (gemcitabine and cisplatin) and ICIs showed promising activity in MIBC, and a randomized phase III trial evaluating perioperative pembrolizumab in combination with GC validated these findings.^[^ ^16^ ^]^ The combination chemo–immunotherapy method was studied in other cancers.^[^ ^17^ ^]^

Recently, a strategy to target biomarkers, comprising functional nucleic acid aptamer–drug conjugates (ApDCs), has been developed, which involves coupling an aptamer to the side chain of a small‐molecule drug via a smart responsive linker, which recognizes biomarkers on the surface of cancer cells specifically and delivers the drug to treat cancer. Related studies have reported that aptamer chemotherapeutic drug Gemcitabine conjugates, such as PTK7 and Gemcitabine, jointly produce PTK7‐GEMs and play a good tumor inhibitory role in bladder cancer.^[^ ^18^ ^]^ The systematic evolution of exponentially enriched ligands (SELEX) technique is used to produce short single‐stranded nucleic acid aptamers, which can carry out high affinity or specific binding with their corresponding ligands,^[^ ^19^, ^20^ ^]^ and it has been widely used in cancer diagnosis, detection, and treatment research.^[^ ^21^, ^22^, ^23^ ^]^ The nucleic acid aptamer is nonimmunogenic, small, has high affinity, good target specificity, is stable and easy to synthesize, and has few side effects.^[^ ^24^, ^25^, ^26^, ^27^, ^28^ ^]^ Therefore, ApDCs not only achieve targeting specificity of anticancer drugs to tumor tissues, but also significantly reduce the problems of excessive loss of anticancer drugs in the blood circulation and excessive toxic side effects to normal tissues and organs.

PD‐L1 aptamers have been reported to have great antitumor efficacy via blocking the binding between PD‐L1 on the cancer cell surface and PD‐1 on the T lymphocyte surface.^[^ ^29^ ^]^ Indeed, bispecific aptamers have been constructed by some investigators with surprising results.^[^ ^30^, ^31^, ^32^ ^]^ The mechanism of these antagonistic DNA aptamers targeting PD‐L1 is similar to that of ICIs. However, there has been no report on the combination of PD‐L1 aptamers and chemotherapeutic drugs for the treatment of bladder cancer.

**Scheme 1:**
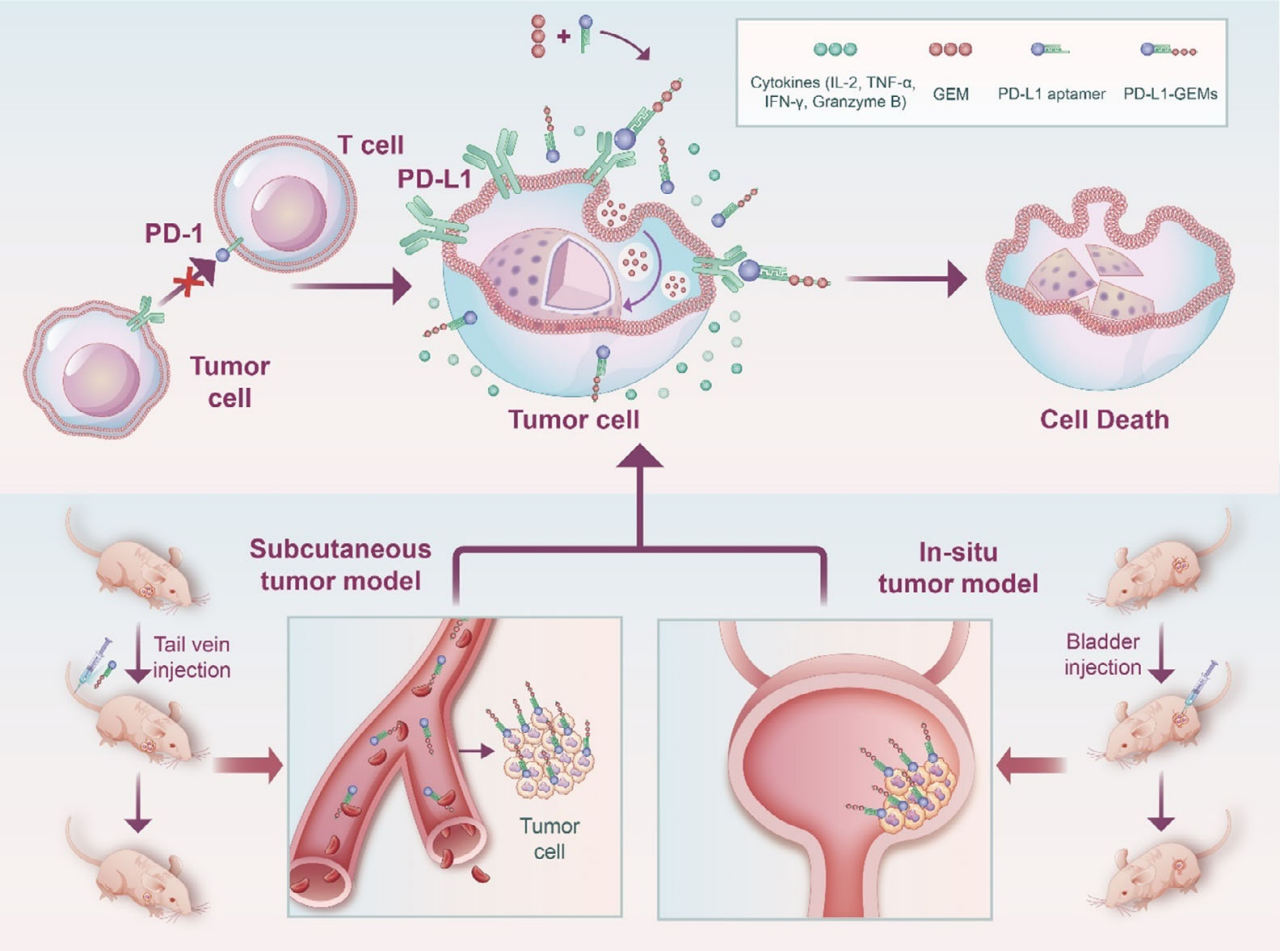
The synthesis and application of PD‐L1‐GEMs in bladder cancer.

Based on the above discussion and findings, we confirmed that PD‐L1 is a biomarker in bladder cancer and is overexpressed in bladder cancer cells. Then, we synthesized a new material for targeting bladder cancer, comprising PD‐L1 aptamers coupled with GEM (PD‐L1‐GEMs) through phosphodiester bonding. This material utilizes the targeting characteristics of the PD‐L1 aptamer to transport GEM to tumor tissue through the blood system and then releases GEM under the action of a phosphatase. The released GEM can be absorbed by bladder cancer cells through endocytosis, to exert apoptosis‐inducing toxic effects. PD‐L1 aptamers can also specifically bind to the PD‐L1 protein on the surface of bladder cancer cells. Competitive binding then inhibits the binding of PD‐1 on the surface of T lymphocytes with PD‐L1, thereby restoring the function of T lymphocytes, promoting the production and aggregation of cytokines and exerting an immune killing role on tumor tissue. In addition, two human tumor xenograft mouse models (subcutaneous tumor and bladder in situ tumor) were used to verify the biosafety and antitumor efficacy of PD‐L1‐GEMs in vivo. (**Scheme** **1**)

## 2 Results

### 2.1 PD‐L1 Expression Upregulated in Bladder Cancer

**Figure 1:**
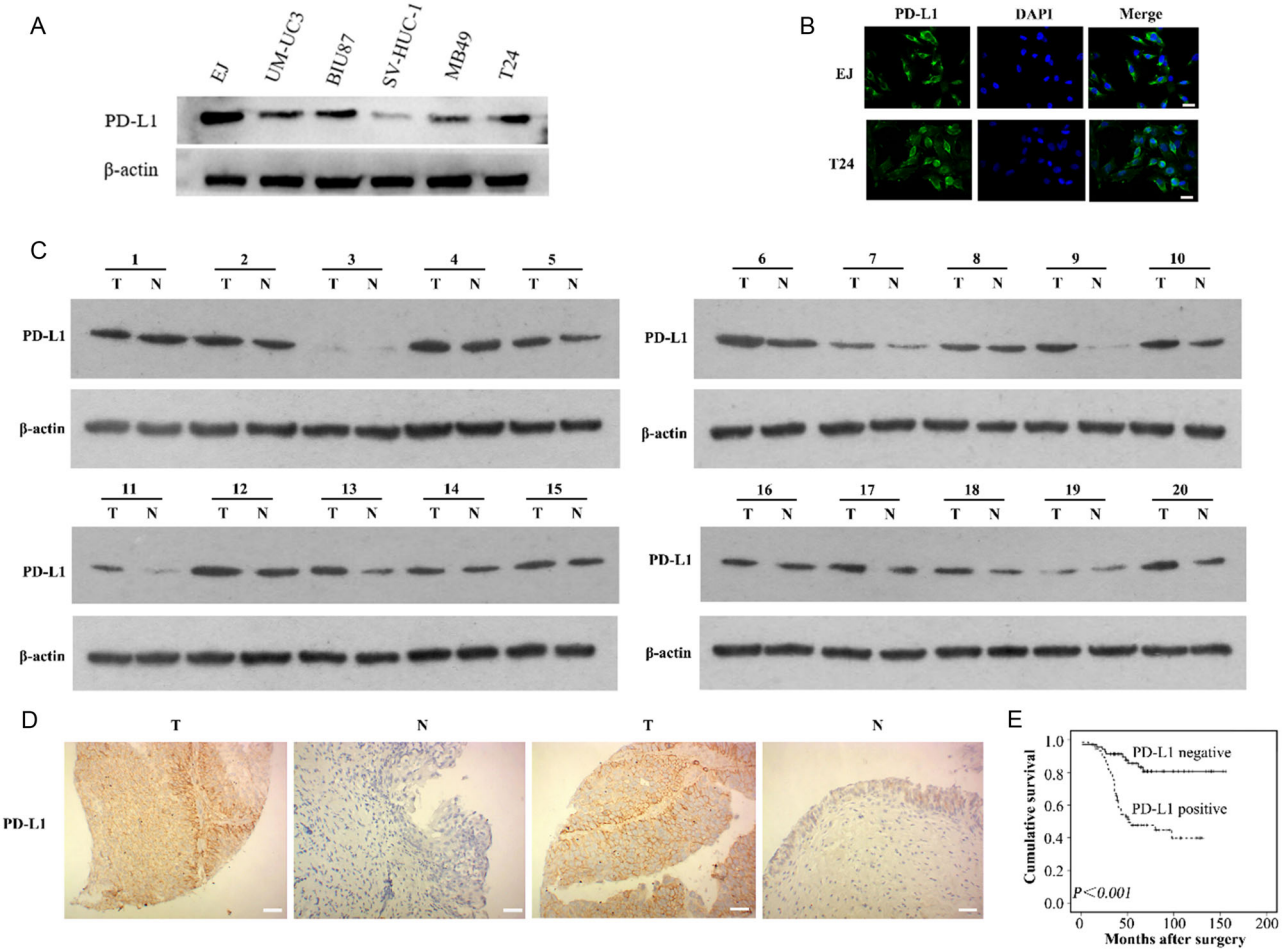
PD‐L1 is upregulated in bladder cancer cell lines and tissues, and this upregulation is associated with poor prognosis. A) PD‐L1 is overexpressed in bladder cancer cell lines compared with normal uroepithelial cell line, SV‐HUC‐1. B) Immunofluorescence showing that PD‐L1 is mainly present in the membrane of bladder cancer cells. Scale bars: 20 μm. C) Western blotting detection of the level of PD‐L1 in surgical specimens of bladder cancer and its adjacent tissues from patients with tumors. D) Representative PD‐L1 immunohistochemical staining in bladder cancer specimens and normal bladder tissue. Scale bars: 100 μm. E) Kaplan–Meier plots of the survival of patients with bladder cancer with positive and negative expression of PD‐L1.

To study the expression of PD‐L1 in bladder cancer, we first detected the expression of PD‐L1 in bladder cancer cells and human normal urothelial cells. The results showed that PD‐L1 was overexpressed in bladder cancer cells compared with that in human normal urothelial cells (SV‐HUC‐1 cells) (**Figure** **1**A). Particularly, EJ and T24 cells had the highest PD‐L1 expression levels. Moreover, the immunofluorescence results showed that specific PD‐L1 protein staining could be observed in the membrane of the bladder cancer cells **(**Figure 1B **)**. Next, we collected 20 surgical specimens of bladder cancer and their adjacent tissues from patients with tumors. The Western blotting results showed higher levels of PD‐L1 in the bladder cancer tissues compared with that in the adjacent tissues of 20 sample pairs (Figure 1C). To further explore the expression of PD‐L1 of bladder cancer, immunohistochemical staining was carried out on 148 bladder cancer tissues and 45 adjacent tissue sections. The results showed that the positive rate of the PD‐L1 expression was 51.35% (76/148) in bladder cancer cells and 13.33% (6/45) in the adjacent bladder normal urothelial cells (*p* < 0.001, Figure 1D).

### 2.2 In Patients with Bladder Cancer, PD‐L1 Expression Correlated with Malignancy and Survival

PD‐L1 levels in the 148 bladder cancer samples were determined using immunohistochemistry (IHC) (Figure 1D), and the relationship between PD‐L1 levels and the patients’ clinicopathological characteristics was determined. As summarized in Table S1, Supporting Information, the PD‐L1 level correlated with the tumor grade, muscularis invasion, and lymph node metastasis (*p* < 0.05). The significance of PD‐L1 in patient prognosis was then assessed. PD‐L1 levels were significantly and negatively associated with the survival of patients with bladder cancer (*p* < 0.05, Figure 1E). Univariate analysis showed that PD‐L1 positive expression was an unfavorable prognostic factor for patients with bladder cancer (*p* < 0.05, Table S2, Supporting Information). Multivariate Cox regression models adjusted for tumor grade, muscularis invasion, lymph node metastasis, showed that the PD‐L1 positive expression was an independent unfavorable prognostic factor for patients with bladder cancer (hazard ratio [HR] 3.122, 95% confidence interval (CI): 1.450–6.725, *p* < 0.05, Table S3, Supporting Information).

### 2.3 Aptamer PD‐L1‐GEMs Synthesis

Commercial GEM and *N,N*‐diisopropylchlorophosphamide were used to synthesize GEM phosphoramidite, with a yield of 80.0% (Figure S1–S4, Supporting Information). The PD‐L1 aptamer was synthesized from the 3′ end to the 5′ end of the oligonucleotide by operating an automated solid‐phase DNA synthesizer (Figure S5–S9, Supporting Information). The PD‐L1‐GEMs were constructed from three GEM phosphoramidite moieties and one PD‐L1 aptamer. The supporting information shows the detailed synthesis method. High‐performance liquid chromatography (HPLC) was used to purify the aptamers, which were identified using mass spectrometry (Figure S10–S17, Supporting Information**)**. Table S4, Supporting Information, shows the sequences of the aptamers.

### 2.4 The Targeting Specificity of PD‐L1‐GEMs toward Bladder Cancer Cells

**Figure 2:**
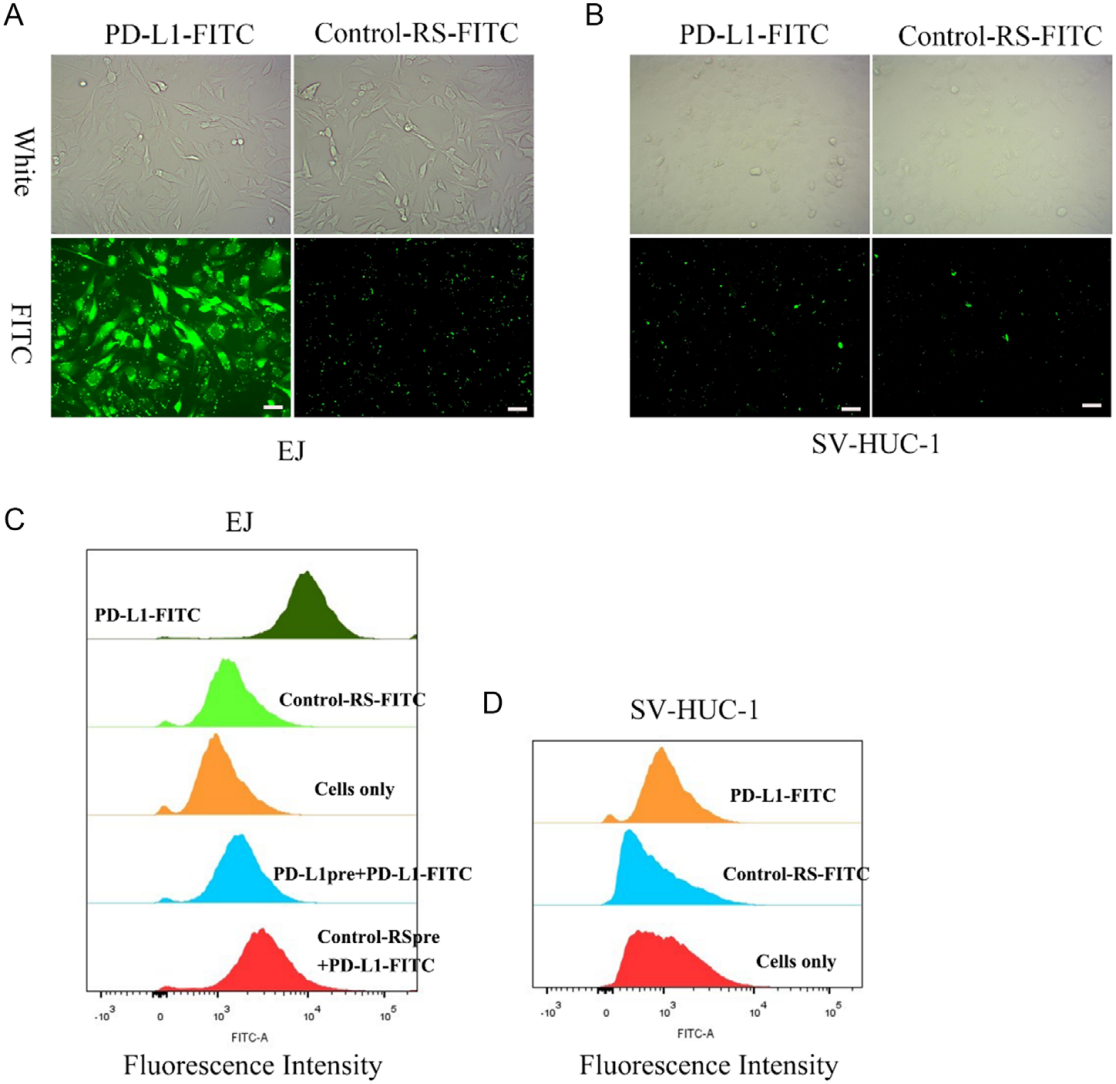
PD‐L1‐GEMs binding specificity and affinity in vitro. A,B) Representative images of FITC‐labeled PD‐L1‐GEMs and Control‐RS‐GEMs‐treated EJ and SV‐HUC‐1 cells. Scale bars: 20 μm. C,D) The targeting ability of FITC‐labeled PD‐L1‐GEMs toward EJ and SV‐HUC‐1 cells.

To assess the targeting specificity of PD‐L1‐GEMs, FITC‐labeled PD‐L1‐GEMs and Control‐RS‐GEMs were incubated with EJ cells as the experimental group, and SV‐HUC‐1 cells formed the negative control group. PD‐L1‐GEMs bound readily to the EJ cell surface; however, no fluorescent signals were detected in EJ cells treated using Control‐RS‐FITC (**Figure** **2**A). No fluorescent signals were observed in both the PD‐L1‐FITC and Control‐RS‐FITC groups in the negative control cell SV‐HUC‐1 cells, indicating that PD L1‐GEMs only bound to cells with high PD‐L1 expression (Figure 2B).

The specificity of bladder cancer cell targeting by PD‐L1‐GEMs was further assessed using flow cytometry. PD‐L1‐GEMs showed a high affinity for EJ cells, while Control‐RS‐GEMs did not bind efficiently to EJ cells. Moreover, SV‐HUC‐1 cells were not targeted by PD‐L1‐GEMs (Figure 2C). Competition experiments further demonstrated that PD‐L1‐GEMs could target EJ cells, and pretreatment with the single PD‐L1 aptamer could prevent PD‐L1 on the surface of EJ cells from binding to PD‐L1‐GEMs (Figure 2D).

### 2.5 The Drug Release and Stability of PD‐L1‐GEMs in Serum

**Figure 3:**
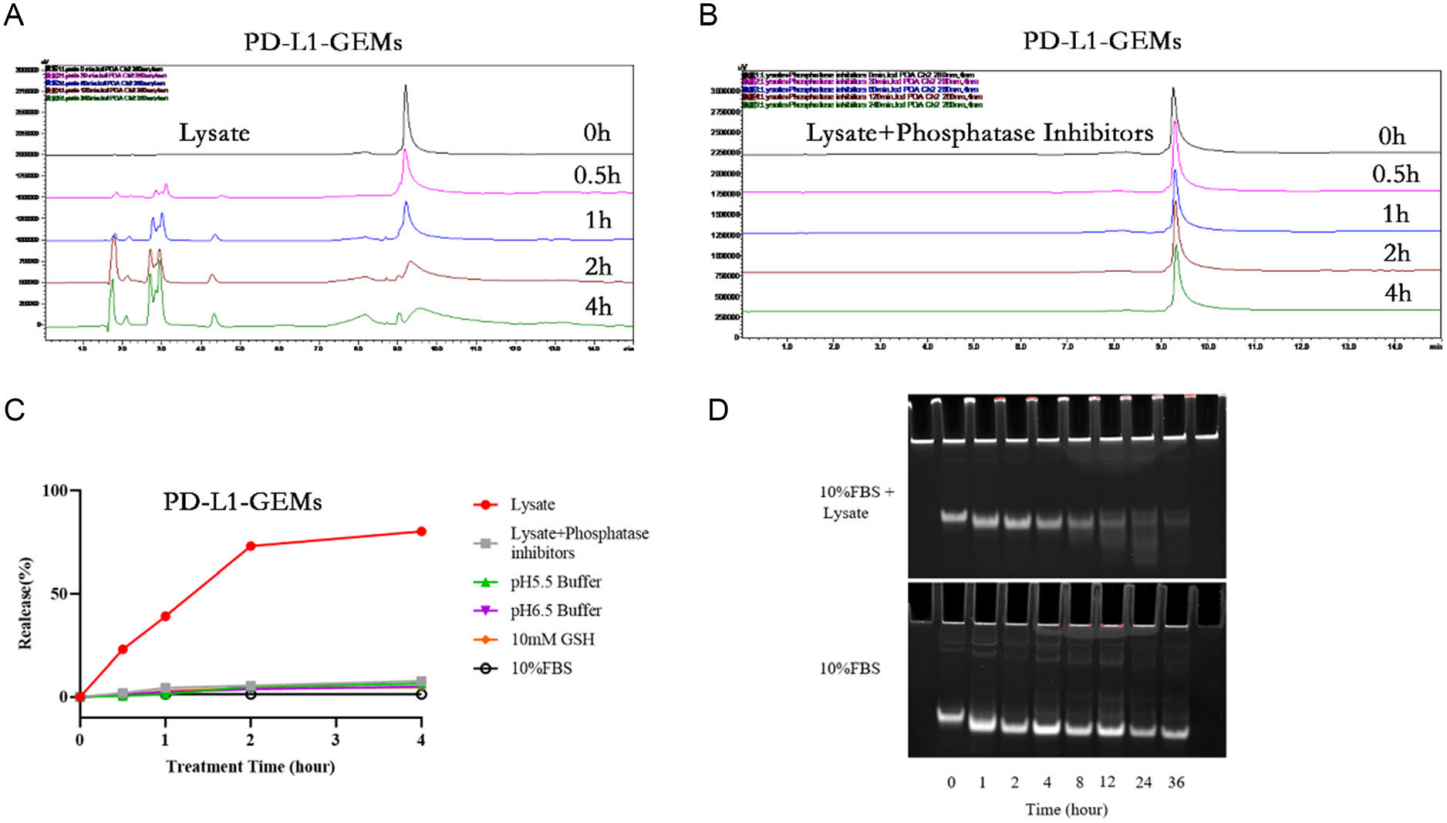
PD‐L1‐GEMs drug release and stability. A,B) The HPLC chromatograms of GEM release from PD‐L1‐GEMs in an EJ cell lysate (untreated by inhibitors) and a phosphatase inhibitor cotreated EJ cell lysate, measured the DNA maximum absorption of 260 nm. C) Cumulative GEM release from PD‐L1‐GEMs incubated with the crude cell lysate, the phosphatase inhibitor‐treated cell lysate, pH 5.5 buffer, pH 6.5 buffer, 10% FBS, and 10 mm GSH, respectively. D) PD‐L1‐GEMs stability in media containing lysate and 10% FBS.

To investigate the mechanism of PD‐L1‐GEMs intracellular release, HPLC analysis of substance release was determined under a variety of conditions, for example, in the cell lysate, lysate added with a phosphatase inhibitor, 10% fetal bovine serum (FBS) (cell culture medium), low‐pH 5.5 buffer (simulating the tumor microenvironment), pH 6.5 buffer (simulating the acid endonuclease/lysosomal system), 10% FBS (cell culture medium), and 1 × 10^−2^ M glutathione (simulating tumor reduction microenvironment). The phosphodiester bond linking PD‐L1 and GEMs in the ApDCs allowed the drug to be released by intracellular phosphatase activity. When PD‐L1‐GEMs (without the inhibitor) were treated with crude cell lysate of EJ cell line at 37 °C, the time‐dependent release of GEM (retention time: 9.2 min) and different sized aptamer fragments (retention time: 1.8, 2.1, 2.7, 3.0, 4.4 min) were observed. About 40% of PD‐L1‐GEMs were degraded within 1 h and almost 80% in 4 h (**Figure** **3**A). The addition of phosphatase inhibitors hindered GEM release from PD‐L1‐GEMs significantly within 4 h (Figure 3B). Of note, PD‐L1‐GEMs incubated under other different conditions showed no significant degradation within 4 h (Figure 3C and S18–S21, Supporting Information). These results demonstrated that phosphatase‐mediated degradation led to GEM release from PD‐L1‐GEMs.

To evaluate the stability of PD‐L1‐GEMs in biological medium, DMEM cell medium supplemented with 10% FBS was used to simulate the tumor cell growth environment. In this medium, the degradation of PD‐L1‐GEMs was not obvious in the first 24 h, and the degradation rate was 14% in 24 h and 22% in 36 h. The control group, which was treated with lysate, already experienced some decomposition starting from 1 h, and by 4 h, the concentration of PD‐L1‐GEMs was already quite low (Figure 3D). Therefore, PD‐L1‐GEMs showed good stability, which could ensure a higher blood drug concentration and increased the opportunity of PD‐L1‐GEMs to act on tumor issue.

### 2.6 Endocytic Pathways of PD‐L1‐GEMs

**Figure 4:**
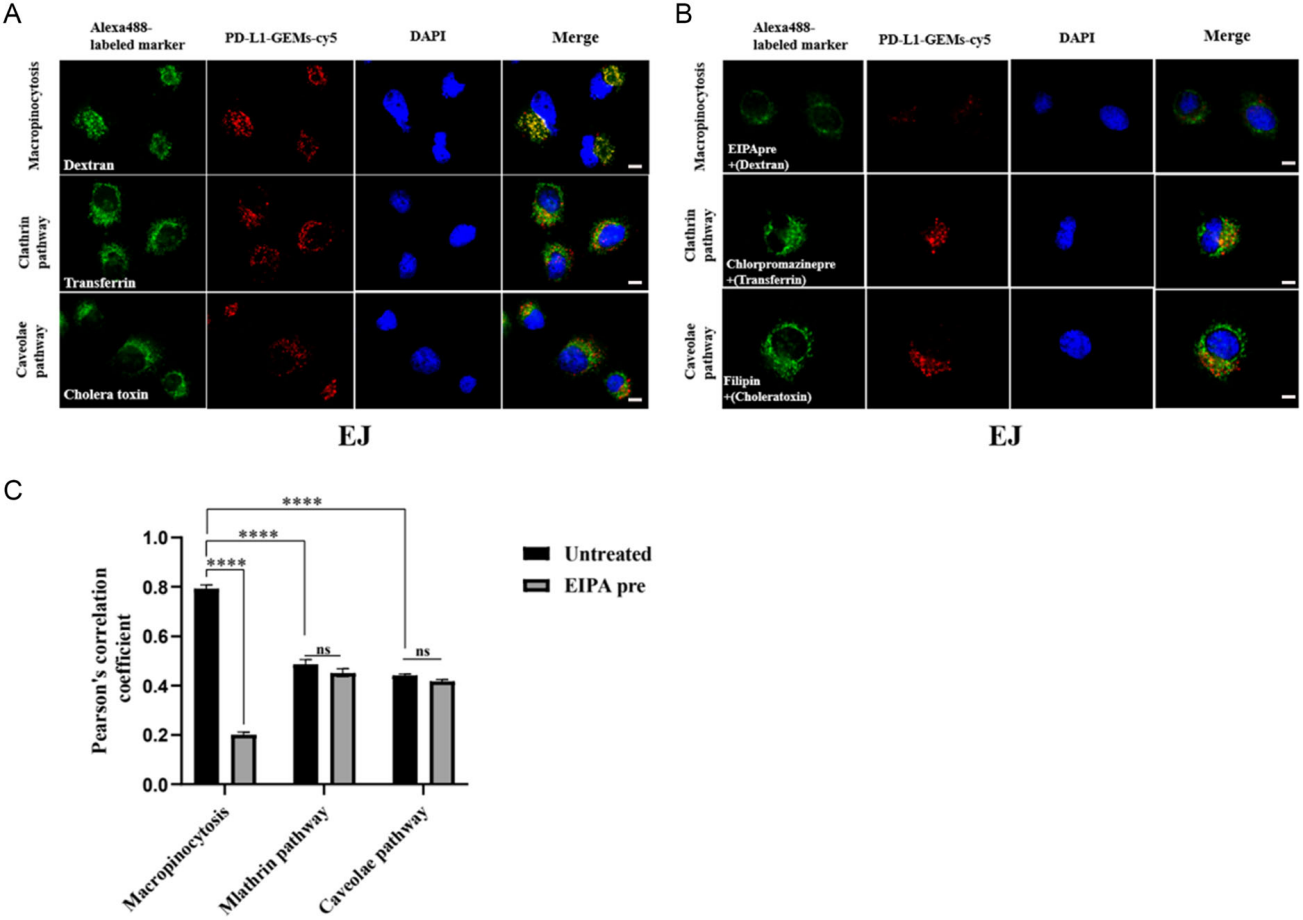
Internalization and trafficking of PD‐L1‐GEMs in bladder cancer cells. A) Confocal microscopy images of PD‐L1‐GEMs‐cy5 (red) colocalization of endocytic markers (dextran, transferrin and cholera toxin) labeled with Alexa Fluor 488. B) The colocalization of dextran, transferrin, and cholera toxin with PD‐L1‐GEMs‐cy5 (red) after EIPA (an inhibitor of macro pinocytosis) pretreatment. Scale bars: 10 μm. C) Image J was used to analyze the Pearson correlation coefficient between PD‐L1‐GEMs‐cy5 and endocytosis markers in EJ cells. Data are shown as the mean ± SEM, *n* = 3. ****p < 0.0001; ns: no significant difference.

To study the uptake pathway of PD‐L1‐GEMs by bladder cancer cells, endocytic markers (cholera toxin, transferrin, and dextran) labeled with Alexa Fluor 488 were used to study EJ cells and the results were observed under a confocal microscope. Dextran (a marker for macropinocytosis, green) and PD‐L1‐GEMs‐cy5 (red) showed significant colocalization. In comparison, PD‐L1‐GEMs‐cy5 did not colocalize with cholera toxin (a marker for caveolae mediated endocytosis) or transferrin (a marker for clathrin‐mediated endocytosis) (**Figure** **4**A). No colocalization of Control‐RS‐GEMs‐cy5 in the EJ cells was observed (Figure S22, Supporting Information). We also conducted a pathway inhibition experiment. EJ cells were preincubated with three different endocytosis inhibitors: EIPA (an inhibitor of micropinocytosis pathway), chlorpromazine (a clathrin pathway inhibitor), and filipin (a caveolae pathway inhibitor). The results showed that the colocalization of PD‐L1‐GEMs‐cy5 and dextran was obviously reduced by EIPA compared with the no inhibitor group. Chlorpromazine and filipin treatment had no significant effect (Figure 4B). Statistical analysis showed that the Pearson correlation coefficient of macropinocytosis was ≈0.8, which decreased to ≈0.2 after EIPA treatment (Figure 4C). The result indicates that the tumor cells with high expression of PD‐L1 take up PD‐L1‐GEMs through macropinocytosis.

### 2.7 The Cytotoxicity of PD‐L1‐GEMs

**Figure 5:**
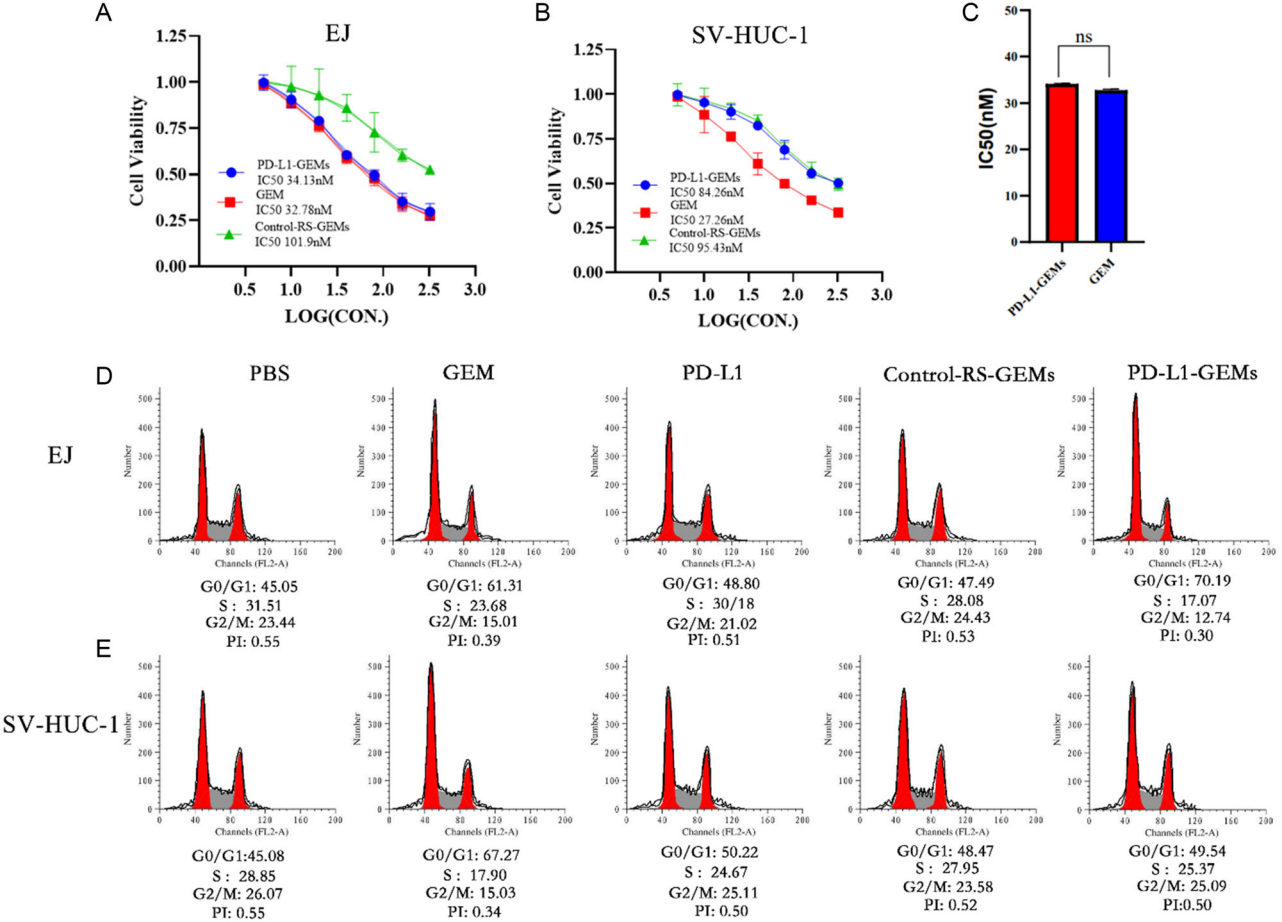
Detection of PD‐L1‐GEMs cytotoxicity and cell cycle analysis. A) The viability of EJ cells and B) SV‐HUC‐1 cells after treatment with PD‐L1‐GEMs, Control‐RS‐GEMs, and GEM, respectively, assessed using CCK‐8 assay (*n* = 3). ns: no significant difference. C) The IC50 of GEM and PD‐L1‐GEMs toward EJ cells. D,E) The cell cycle in EJ cells and SV‐HUC‐1 cells after treatment with PD‐L1‐GEMs, Control‐RS‐GEMs, GEMs, PD‐L1, and blank surface control, as detected using flow cytometry. (*n* = 3). ns: no significant difference.

The cytotoxicity of PD‐L1‐GEMs toward bladder cancer EJ cells and normal bladder epithelial cell SV‐HUC‐1 cells was assessed using Cell Counting Kit‐8 (CCK‐8) assay. GEM has no selective specificity for EJ cells or SV‐HUC‐1 cells; therefore, its toxic effects toward the two types of cells are similar. PD‐L1‐GEMs had a similar toxic effect on EJ cells as GEM, but a weaker toxic effect on SV‐HUC‐1 cells, similar to that of Control‐RS‐GEMs. (**Figure** **5**A–C). This result mainly reflected the targeting specificity of PD‐L1‐GEMs on bladder cancer EJ cells, and GEM without this feature would induce serious side effects. Similar results were observed in the flow cytometry analysis of the cell cycle and apoptosis. PD‐L1‐GEMs increased the G1/S phase ratio of EJ cells compared with GEM, but Control‐RS‐GEMs showed the similar effect as the blank control group. However, there was no significant difference of PD‐L1‐GEMs for SV‐HUC‐1 cells compared with blank control group and Control‐RS‐GEMs group (Figure 5D,E, and S23, Supporting Information). These results showed that PD‐L1‐GEMs exert a strong toxic effect on bladder cancer cells and reduce the toxicity due to the binding specificity of PD‐L1 aptamer to the PD‐L1 target.

### 2.8 Biological Imaging and PD‐L1‐GEMs’ Antitumor Activity in Xenotransplanted Subcutaneous Tumors

**Figure 6:**
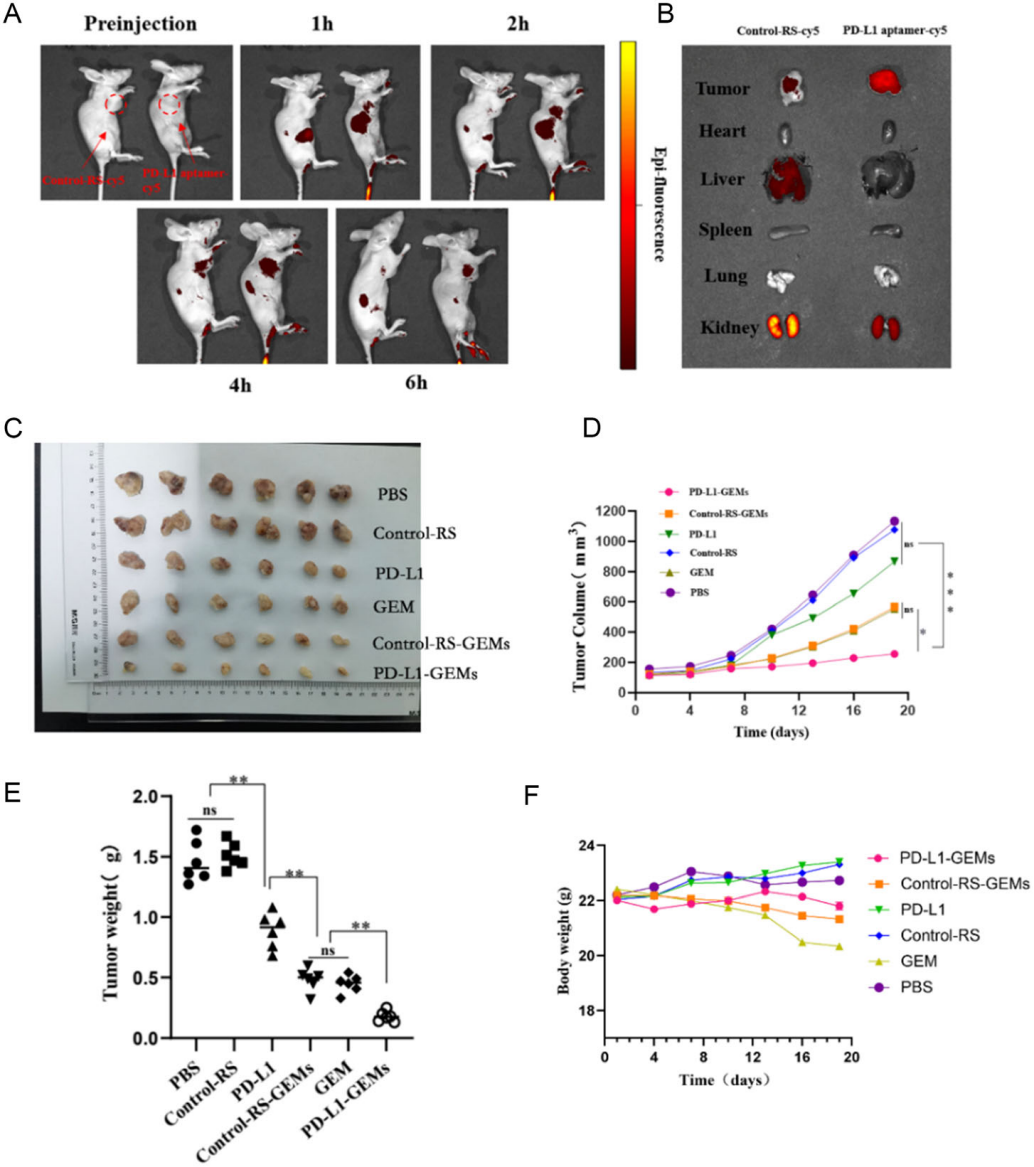
PD‐L1‐GEMs biological imaging and antitumor activity in xenotransplanted subcutaneous tumors. A) Bioimaging of PD‐L1‐GEMs‐cy5 (right) and Control‐RS‐GEMs‐cy5 (left) at 0,1, 2, 4, and 6 h postcaudal vein injection in the subcutaneous tumor model. B) PD‐L1‐GEMs‐cy5 and Control‐RS‐GEMs‐cy5 biodistribution in major organs and tumors at 6 h postinjection. C) Post‐treatment comparison of tumor tissues from the six groups. D) Growth curves of tumors receiving different treatments. Changes in E) tumor weight and F) body weight among the groups of xenografted mice. Data shown are the mean ± SD; *p < 0.05; **p < 0.01, ***p < 0.001; (*n* = 6). ns: no significant difference.

To verify that PD‐L1‐GEMs‐cy5 targeted bladder cancer in vivo, the tumor tissue distribution of PD‐L1‐GEMs‐cy5 in EJ cell xenograft tumor models was measured using living imaging technology. PD‐L1‐GEMs‐cy5 treatment resulted in a stronger fluorescence intensity than that in mice treated with Control‐RS‐GEMs‐cy5. The PD‐L1‐GEMs‐cy5 fluorescence accumulated rapidly, reaching a peak at 4 h, and the intensity gradually decreased, but was still detectable, after 6 h. (**Figure** **6**A). The tumors and major organs were dissected from sacrificed nude mice and used for optical imaging. The tumors treated with PD‐L1‐GEMs‐cy5 had strong fluorescence intensity; however, the fluorescence signal in corresponding major organs was weak (Figure 6B). These results demonstrated that PD‐L1‐GEMs‐cy5 has a good tumor targeting ability.

The effect of PD‐L1‐GEMs on tumor inhibition was assessed in the xenograft subcutaneous tumor model. The data showed that the PD‐L1‐GEMs were more effective than the Control‐RS‐GEMs and GEM at inhibiting tumor growth, reducing the tumor volume and reducing tumor quality. Based on the analysis of the tumor volume, tumor weight, and mouse body weight, the PBS group and the Control‐RS group showed the largest increase in tumor volume, indicating that they did not have a therapeutic effect. PD‐L1‐GEMs, Control‐RS‐GEMs, and GEM treatments significantly reduced the tumor volume. Compared with the tumor volume of the PBS group, the tumor volume of the GEM and Control‐RS‐GEMs groups decreased by 48.6% and 41.6%, respectively, while that of the PD‐L1‐GEMs group decreased by ≈74.6% (Figure 6C–E), which indicated that because of the advantages of PD‐L1‐GEMs in targeting tumor tissue characteristics, the inhibitory effect of GEM on tumor growth was greatly enhanced. In addition, the weight of the mice in the GEM and Control‐RS‐GEMs groups decreased by 9.2% and 3.9%, respectively, while no significant changes were found in the PD‐L1‐GEMs group and the other three groups (Figure 6F). These results fully demonstrated the advantages of the PD‐L1 aptamer as a carrier for chemotherapeutic drugs for targeted treatment.

Besides, Ki‐67 staining showed that the proliferation of bladder cancer cells in PD‐L1‐GEMs treatment group was the lowest, indicating that the inhibitory effects of PD‐L1‐GEMs toward the bladder cancer cells was the strongest (Figure S24A, Supporting Information). TUNEL assays and PD‐L1 staining suggested higher levels of apoptosis, cell death, and PD‐L1 infiltration in the PD‐L1‐GEMs treatment group compared with those in the other groups (Figure S24B,C, Supporting Information). Moreover, we further analyzed lymphocyte infiltration and cytokine secretion in the mouse tumors. Lymphocyte infiltration and the levels of cytokines (IL‐2, TNF‐α, IFN‐γ, and granzyme B) increased markedly in the PD‐L1‐GEMs and PD‐L1 groups, whereas the other four groups had very low levels of infiltration or cytokines (Figure S25, Supporting Information). These data indicated that targeted delivery of GEMs by PD‐L1‐GEMs not only exerted a chemotherapeutic effect, but also enhanced the immune response, thus playing a dual therapeutic role.

### 2.9 PD‐L1‐GEMs’ Antitumor Activity in the Bladder Cancer In Situ Model

**Figure 7:**
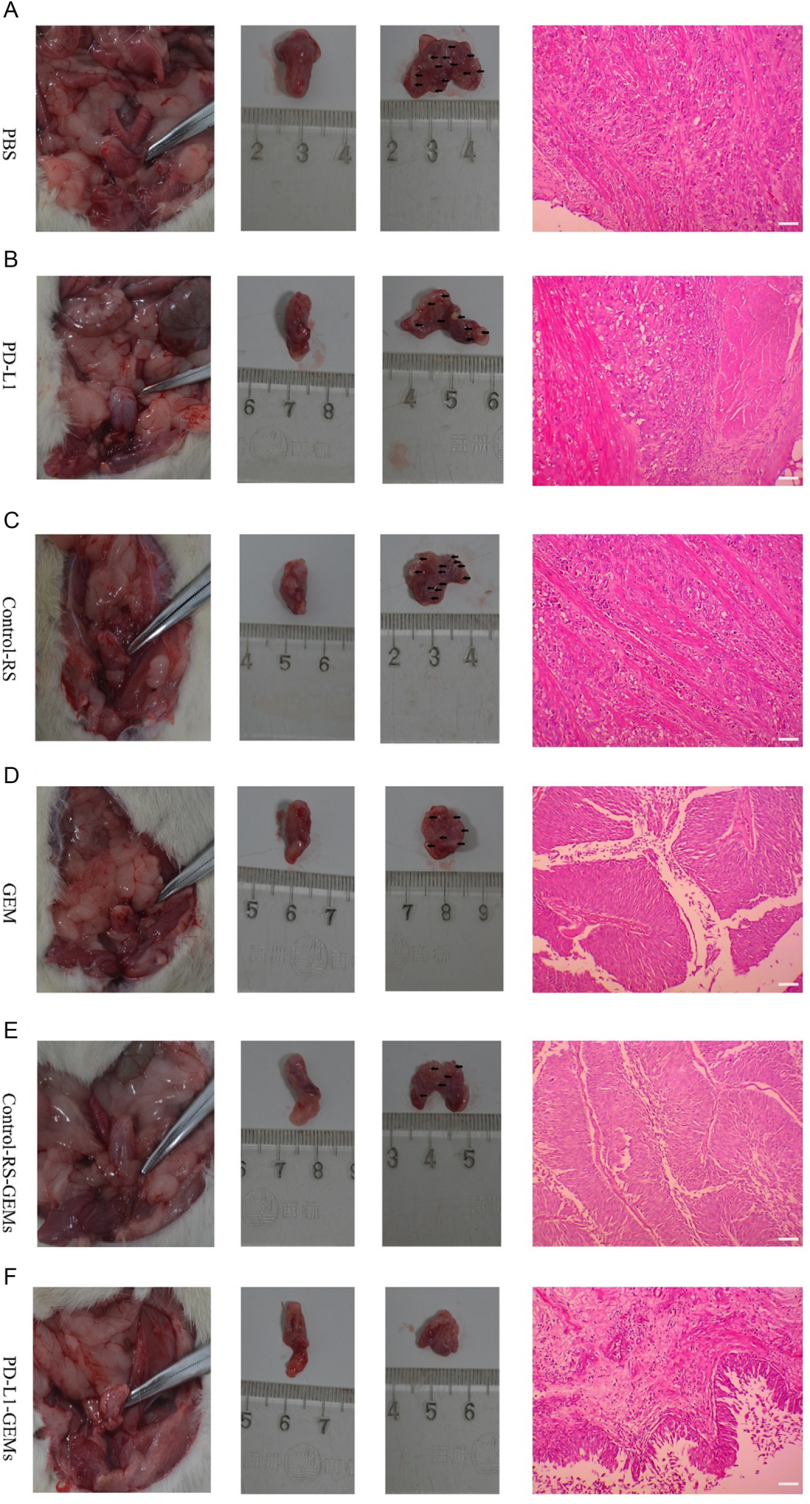
PD‐L1‐GEMs’ antitumor effect in an in situ bladder cancer model. Representative images of excised bladders and tissue sections stained using H&E and treated with A) PBS (tumors are muscle‐invasive bladder cancer: ≥ stage pT2), B) PD‐L1 (tumors are muscle‐invasive bladder cancer: stage pT2), C) Control‐RS (tumors are muscle‐invasive bladder cancer: ≥stage pT2), D) GEM (tumors are noninvasive papillary carcinoma: stage pT1), E) Control‐RS‐GEMs (tumors are noninvasive papillary carcinoma: stage pT1), and F) PD‐L1‐GEMs (tumors are noninvasive papillary carcinoma: stage ≤ pTa). Scale bar, 100 μm.

Urinary bladder instillation chemotherapy is commonly used to treat bladder cancer. Therefore, the clinical significance of PD‐L1‐GEMs was assessed by determining their tumor suppressive effect in a bladder cancer in situ model. The changes in bladder histopathology in the PD‐L1‐GEMs group were significantly different compared with those in the other treatment groups (Table S5, Supporting Information). Interestingly, in most of the examined bladder cancers, PD‐L1‐GEMs treatment resulted in the lowest tumor stage (stage pT0/Tis/Ta), whereas, in the other groups, the tumor stage was higher than pT2. This result was confirmed by histological examination of excised bladders and H&E staining of tissue slices (**Figure** **7**).

### 2.10 In Vivo Biosafety Assessment of PD‐L1‐GEMs

Biochemical analysis was used to assess the biosafety of PD‐L1‐GEMs in whole blood. The tests included routine blood biochemical analysis and liver and kidney function tests, such as blood cell counts, alanine aminotransferase (ALT), aspartate aminotransferase (AST), and creatinine. The data showed that the PD‐L1‐GEMs group had significantly lower levels of ALT and AST than the GEM and Control‐RS‐GEMs groups, and the ALT and AST levels of the PD‐L1‐GEMs group were similar to those of the PBS group. This indicated that the GEM and Control‐RS‐GEMs groups had obvious hepatotoxicity. Besides, the levels of white blood cells (WBCs) in the GEM and Control‐RS‐GEMs group were decreased compared with those in the PBS and PD‐L1‐GEMs groups. Notably, there were no differences among the groups for red blood cells (RBCs), platelets (PLTs), hemoglobin (HGB), UREA, creatinine (CR), and creatine phosphokinase (CK). Therefore, PD‐L1‐GEMs could significantly reduce the toxicity and side effects of GEM on the liver and showed improved biological safety (Figure S26 and S27 and Table S6, Supporting Information).

Next, the damage caused by GEM to major organs was assessed using H&E staining. The results showed that the liver tissue structure in GEM group and Control‐RS‐GEMs had a different degree of damage. However, the results of in the PBS and PD‐L1‐GEMs group were opposite, which suggested that PD‐L1‐GEMs could effectively reduce the whole body and major organ toxicity caused by GEM (Figure S28, Supporting Information).

## 3 Discussion

Herein, we report a novel material, PD‐L1‐GEMs, formed by combining PD‐L1 aptamer with the chemotherapeutic drug gemcitabine. We found that the PD‐L1‐GEMs could inhibit the growth and progression of tumors, while synergistically enhancing the chemotherapeutic effect of GEM, reducing its serious side effects and simultaneously playing an immunosuppressive role.

Chemotherapy has always been the main treatment method for bladder cancer. GEM is a main first‐line chemotherapy drug whose excellent tumor inhibition effect means that it is used widely in the clinical treatment of bladder cancer.^[^ ^33^ ^]^ However, because of its poor stability, chemotherapy‐induced drug resistance, and lack of targeting ability, GEM causes adverse effects. Researchers developed more treatment options, such as immunotherapy, gene therapy, and the combination of chemotherapy and immunotherapy; however, some results are unsatisfactory. Therefore, in this study, we aimed to use new methods to enhance the chemotherapy efficacy of GEM and reduce its severe toxic side effects.

Recent studies have noted that PD‐L1 is highly expressed in several tumor types, such as colorectal cancer,^[^ ^34^ ^]^ lung cancer,^[^ ^35^ ^]^ glioblastoma,^[^ ^36^ ^]^ breast cancer,^[^ ^37^ ^]^ and ovarian cancer.^[^ ^38^ ^]^ We showed that PD‐L1 is also overexpressed in bladder cancer and confirmed that it could be used as a biomarker, which was consistent with previous studies.^[^ ^39^ ^]^ PD‐L1 binds to PD‐1 on the surface of T lymphocytes, sending out inhibitory signals and reducing the proliferation of T lymphocytes, and is thus related to immunosuppression.^[^ ^40^ ^]^ Researchers have utilized the characteristics of PD‐L1 to design single aptamers, dual aptamers, and ICIs^[^ ^41^, ^42^ ^]^ and have achieved good research feedback. Therefore, the recognition of the dual identity of PD‐L1 represents a breakthrough in the treatment of bladder cancer.

Single‐agent chemotherapy often does not meet the requirements of patients, even though they receive aggressive treatments (neoadjuvant chemotherapy). Indeed, more than 40% of patients with MIBC still experienced recurrence or death within 3 years,^[^ ^43^, ^44^, ^45^ ^]^ similar to those receiving immunotherapy. Surprisingly, the application of chemotherapy combined with immunotherapy (ICIs) has become a success in several carcinomas, which could result in superior survival benefits compared with chemotherapy or immunotherapy alone.^[^ ^46^, ^47^, ^48^ ^]^ Therefore, we attempted to connect PD‐L1 with GEM to achieve our research objective in the treatment of bladder cancer.

Accordingly, we designed a PD‐L1 aptamer after determining that PD‐L1 is highly expressed in bladder cancer and then combined three gemcitabine molecules with each aptamer through a phosphodiester bond to increase the efficiency of drug delivery. Experiments showed that PD‐L1‐GEMs have good stability during blood transportation, greatly reducing drug loss. Specific binding analysis showed that PD‐L1‐GEMs could accurately deliver GEM to tumor tissue, suggesting a significant improvement in the utilization efficiency of GEM and a reduction in toxic side effects on related organs. After PD‐L1‐GEMs targeted tumor cells, under the action of a phosphatase, free GEM is released free, which is ingested through the macrocytosis pathway to induce cytotoxicity. Moreover, the low affinity of PD‐L1‐GEMs toward normal epithelial cells, means that PD‐L1‐GEMs have lower cytotoxicity than chemotherapy alone. Biological imaging also indicated that PD‐L1‐GEMs have significant targeting specificity for tumor tissues. Moreover, the biosafety and antitumor efficacy of PD‐L1‐GEMs were validated by constructing subcutaneous tumorigenesis and bladder in situ cancer models in vivo. As expected, the PD‐L1‐GEMs showed the most significant inhibitory effect on tumor growth and progression among the treatments. Meanwhile, we found that the infiltration of T lymphocytes and secretion of cytokines in tumor tissue increased, which indicated an active immune response. This indicated that PD‐L1‐GEMs could simultaneously play a role in targeted chemotherapy and immunotherapy to treat bladder cancer.

## 4 Conclusion

In conclusion, we synthesized a novel targeted material, PD‐L1‐GEMs, by aptamer–drug conjugation. Furthermore, the in vivo antitumor efficiency and biosafety of PD‐L1‐GEMs were evaluated using two mouse models of tumor transplantation. These experimental results indicated that PD‐L1‐GEMs represent breakthrough in the treatment of bladder cancer. We believe that this combination of aptamers and drugs has good application prospects for the targeted treatment of bladder cancer.

## 5 Experimental Section

5.1

5.1.1

##### Reagents

Abcam (Cambridge, MA, USA) provided the rabbit monoclonal antibody recognizing PD‐L1. Beyotime Biotech Inc (Jiangsu, China) provided the annexin V‐FITC Apoptosis Detection Kit (no. 011 821 210 207), the protease inhibitors, the phosphatase inhibitors cocktail, radioimmunoprecipitation assay (RIPA) lysis buffer, lysotracker, Hoechst 33 342, and the goat antimouse secondary antibody labeled with Alexa Fluor 488. Sangon Biotech Co. Ltd. (Shanghai, China) provided the protein markers and US Everbright Inc (Sayreville, NJ, USA) provided Super GelRed. Beijing Labor Technology Co, Ltd (Beijing, China) provided the Cell Counting Kit‐8 (CCK‐8). Energy Chemical (Shanghai, China) supplied all the chemical used for synthesis and purification. NEST Biotechnology (Jiangsu, China) supplied the cell culture tubes and plates. All reagents not mentioned above were supplied by Sigma‐Aldrich (St. Louis, MO, USA).

##### Cells Culture

Cell lines derived from bladder cancer (EJ, T24, UM‐UC3, BIU87, MB49) and the normal cell line (SV‐HUC‐1) were purchased from the ATCC (Manassas, VA, USA). EJ, T24, UM‐UC3, BIU87, and MB49 cells were cultured in Dulbecco's modified Eagle's medium (DMEM), whereas SV‐HUC‐1 cells were cultured in F12 medium. The above medium was added with 10% FBS, 1% glutamine, and 1% penicillin–streptomycin. All cells were cultured in a humidified atmosphere of 5% CO_2_ at 37 °C.

##### Clinical Bladder Cancer Specimens and Patients

We collected bladder tissue samples from 148 patients (148 bladder cancer samples and 45 adjacent non tumorous tissues) who had detailed clinical information and received surgery between 2002 and 2012 at the Third Xiangya Hospital of Central South University and Sun Yat‐sen University Cancer Center.^[^ ^49^ ^]^ In addition, we collected 20 fresh cancer specimens and paired adjacent noncancerous tissues from patients with primary bladder cancer attending the Third Xiangya Hospital of Central South University. Immediately after surgery, the fresh specimens were snap frozen in liquid nitrogen.

##### Immunofluorescence

Cells in six well plates were washed using 1 × phosphate‐buffered saline (PBS) solution for 5 min, fixed using 4% paraformaldehyde, and allowed to dry naturally. The cells were washed using 1 × PBS buffer three times for 5 min each time, added with normal goat serum blocking solution, and incubated at room temperature for 20 min. The excess liquid was removed, 50 μL of the anti‐PD‐L1 was added, and the cells were incubated overnight at 4 °C. After washing with PBS three times for 5 min each time, the secondary antibody conjugated to Alexa Fluor 488 Goat Anti‐Rabbit IgG (H + L) (0.5 mg mL^−1^ A23220, dilution 1:100) was added to the cells and incubated at 37 °C for 40 min. After washing with PBS three times for 5 min each time, the nuclei were stained by adding 100 μL of 4',6‐diamidino‐2‐phenylindole (DAPI) and incubating at room temperature for 5 min. After washing with PBS three times for 5 min each time, a Leica TCS‐SP5 laser confocal scanning microscope (Leica, Wetzlar, Germany) was used to observe and image the cells.

##### Immunohistochemistry (IHC)

Tumor tissues were embedded in paraffin and then sectioned at 4 μm thick. To evaluate PD‐L1 using IHC, the sections were mounted on glass slides and treated using an automated immunostainer (BenchMark Ultra, Ventana Diagnostic Systems, Tucson, AZ, USA). In brief, antigen retrieval was carried out for 30 min by incubation in Ventana CCI retrieval buffer. Rabbit antihuman PD‐L1 monoclonal antibodies (SP263, Ventana Medical Systems, Inc., Tucson, AZ, USA) were incubated with the sections for 30 min. An OptiView DAB detection kit (Ventana Medical Systems) was used to detect the formed immune complexes. Bladder cancer cell expression of PD‐L1 was then assessed as described previously.^[^ ^50^, ^51^ ^]^ Two investigators who were blinded to the clinical data reviewed the immunostained sections. Any disagreements were resolved via consensus.

##### Western Blotting

Bead milling was used to homogenize the tissues at 4 °C for 5 min, followed by lysis buffer treatment on ice for 30 min. Centrifugation for 30 min at 4 °C and 1000 g was used to collect the proteins. Approximately 50 μg of proteins were subjected to denaturation, 12% SDS/PAGE separation, and transfered onto a polyvinylidene fluoride (PVDF) membrane (LC2002, Invitrogen; Thermo Fisher Scientific, Waltham MA, USA). Then, 5% skimmed milk was used to block nonspecific binding to the membranes, followed by separate incubations with primary antibodies recognizing PD‐L1 (1:2000, Abcam) and β‐actin (Sigma‐Aldrich; 1:2000) at 4 °C for more than 8 h. Subsequently, the membranes were incubated with anti‐rabbit IgG antibody (1:5000, Cell Signaling Technology, Danvers, MA, USA) and anti‐mouse IgG antibody (Thermo Fisher Scientific) for 2 h at room temperature. Finally, the ECL chemiluminescent Substrate (Thermo Fisher Scientific) was used to detect the immunoreactive proteins on the membrane, followed by ImageQuant ECL Imager (GE Healthcare, Chicago, IL, USA) analysis.

##### Binding Specificity Assay

After 0.02% EDTA digestion of EJ and SV‐HUC‐1 cells, about 5 × 10^5^ cells in each group were incubated on ice for 1 h in 200 μL of binding buffer containing 500 nm PD‐L1‐FITC, Control‐RS‐FITC, and cells only (binding buffer containing 10% FBS). The binding buffer consisted of 0.1 mg mL^−1^ tRNA (Sigma), 1 mg mL^−1^ BSA(Sigma), and 5 nm MgCl2. For the competitive combination test, the PD‐L1 aptamers and Control‐RS were preincubated at a concentration of 2.5 μm on ice for 1 h. Each group was washed thrice using 700 μL of PBS and then suspended in 300 μL PBS for flow cytometry detection using the BD FACSVerse system (San Jose, CA, USA).

##### Polyacrylamide Gel Electrophoresis

PD‐L1‐GEMs were incubated in DMEM containing 10% FBS at 37 °C for 0, 1, 2, 4, 8, 12, 24, and 36 h. The samples were then denatured in for 5 min at 95 °C, followed by 12% polyacrylamide gel electrophoresis for 30 min at 110 V. The gel was then stained for 10 min using Super GelRed (US Everbright Inc), followed by imaging.

##### HPLC

To assess the release of gemcitabine in the cell lysate, cells were seeded into a 10 cm large dish in advance, and then added with RIPA cell lysate buffer, with or without phosphatase inhibitor. After sufficient lysis, the cell lysate was treated with 100 μm PD‐L1‐GEMs s for 0, 0.5, 1, 2, and 4 h in a 37 °C constant temperature water bath and then added with acetonitrile in proportion. After fully mixing, the cells were centrifuged in a low‐temperature high‐speed centrifuge for 10 min at 4 °C and 12 000 r min^−1^ and the supernatant was retained. The absorbance of the supernatant at 260 nm was determined using HPLC. In the other groups. PD‐L1‐GEMs were added into buffer at pH 5.5, pH6.5, 10% FBS, or GSH, respectively; the concentration of PD‐L1‐GEMs was 100 μm, which was detected according to the above method. To eliminate the influence of other factors on the experiment, the above experiments were purified to eliminate interference.

##### Endocytic Pathway Analysis

EJ cells were seeded into a 15 mm confocal dish overnight to allow the cells to adhere to the wall. In the noninhibitor group, after blocking the cells with 5% bovine serum albumin (BSA) for 1 h, 200 nm PD‐L1‐GEMs were incubated with the Alexa fluor 488‐labeled endocytic marker (5 μg mL^−1^ transferrin, 25 μg mL^−1^ cholera toxin, and 25 μg mL^−1^ dextran) for 2 h, and then 2 μg mL^−1^ Hoechst 33 342 was added. The cells were rinsed using washing buffer and observed under a confocal microscope. The drug inhibitor group was preincubated with 0.1 mm chlorpromazine (for the clathrin pathway), 1 mm filipin (for the caveolae pathway), and 0.1 mm 5‐[N‐ethyl‐N‐isopropyl] amiloride (EIPA) (for the macropinocytosis pathway) for 2 h, and then 200 nm cy5‐labeled PD‐L1‐GEMs and Alexa fluor 488‐tagged endocytosis markers were added, incubated for 2 h in the presence of inhibitors, and then the cells were washed and observed under a confocal microscope.

##### Cell Apoptosis Assay

After digesting SV‐HUC‐1 and EJ cells in a large dish with 0.02% EDTA, 5 × 10^3^ cells were seeded into a 96‐well plate and treated with PD‐L1‐GEMs, Control‐RS‐GEMs, PD‐L1, Control‐RS, GEM, and PBS respectively for a suitable time. After the intervention, an Annexin V‐PE/7‐AAD cell apoptosis detection kit (Beyotime) was used to stain the cells, followed by detection using flow cytometry.

##### CCK‐8 Assay

The cells were inoculated in 96‐well plates (5 × 10^3^ cells well^−1^) and cultured for 24 h to allow the cells to adhere to the well. Then, 100 μL of a concentration gradient (0, 10, 20, 40, 80, 160, 320 nm) of treatments (PD‐L1‐GEMs, Control‐RS‐GEMs, GEM, PD‐L1, Control‐RS, and PBS) were added to the wells and incubated for 8 h, followed by PBS washing. Fresh, complete DMEM medium was then added and incubated for 72 h. When the predetermined time was reached, 10 μL of CCK‐8 reagent was added to each well and incubated for the 2 h. The absorbance was then measured at 450 nm in a Synergy 2 Multi‐Mode Microplate Reader (Bio‐Tek, Winooski, VT, USA) to determine cell viability.

##### Cell Cycle Analysis

EJ Cells were treated with PD‐L1‐GEMs, Control‐RS‐GEMs, GEM, PD‐L1, Control‐RS, and PBS (about 2 × 10^6^ cells group^−1^) and washed once by PBS, centrifuged at 1000 rpm for 5 min, fixed in precooled 75% ethanol, and incubated overnight at 4 °C. Then, the 75% ethanol was discarded, the cells were washed once in PBS, suspended in 800 μL of PBS and 1% BSA solution, and 100 μL of propidium iodide (PI) dye solution (3.8 × 10^−2^ m sodium citrate, pH 7.0), plus100 μL RnaseA (10 mg mL) was added, followed by incubation for 30 min at 37 °C in the dark. Finally, the cells were assessed using flow cytometry (Beckman Instruments, Inc., Brea, CA, USA).

##### Bioimaging of PD‐L1 Aptamers in vivo

When the volume of the subcutaneous transplanted tumor in the nude mice reached >500 mm^3^, PD‐L1 aptamer‐cy5 and Control‐RS‐cy5 (50 μm, 200 μL) were injected into the tail vein of the mice, separately. After anesthesia using isoflurane, the tumor‐bearing mice were imaged before injection, and at 0, 1, 2, 4, and 6 h after injection, using an IVIS luminaxr optical imaging system (PerkinElmer, Waltham, Ma, USA). At 4 h after injection, the nude mice were sacrificed, and the lung, spleen, liver, heart and tumors were dissected for tissue imaging. The nude mice were sacrificed in a manner that minimized or eliminated pain and distress. All the animal experiments were approved by the Ethics Committee of the Third Xiangya Hospital of Central South University.

##### Antitumor Activity of PD‐L1‐GEMs in Xenotransplanted Subcutaneous Tumors

About 8 × 10^6^ MB49 cells were subcutaneously injected into the right armpit of each BALB/c female mouse. When the tumor grew to about 100–120 mm^3^, the mice were divided into six groups (*n* = 6 per group). Each group was given an equal dose of GEM (equivalent GEM concentration = 16 mg kg^−1^) intravenously, including the PD‐L1‐GEMs, Control‐RS‐GEMs, GEM, PD‐L1, Control‐RS, and PBS (blank control) groups. Each injection was 200 μL and was administered once every two days, for a total of six injections. Before each injection, the body weight and tumor volume of the mice were recorded. At 48 h after the last treatment, the mice were humanely sacrificed and their venous blood was collected for blood biochemistry and liver and kidney function tests. We excised the tumor tissue and dissected out kidney, lung, spleen, liver, and heart and subjected them to IHC staining.

##### Tumor‐Infiltrating Lymphocytes and Secreted Cytokine Analysis

The mice (*n* = 6) were sacrificed at 2 days after the last injection. Excised tumors were used to prepare single‐cell suspensions for analysis using flow cytometry. Tumor cells were labeled using anti‐CD4‐Allophycocyanin (APC) and anti‐CD8‐Phycoerythrin (PE) for quantitative T cell analysis. To assay cytokines (interleukin 2 (IL‐2), interferon gamma (IFN‐γ), tumor necrosis factor alpha (TNF‐α), and Granzyme B), a portion of the tumor from each group was weighed and the homogenized in 1 mL PBS using an automatic sample rapid grinding machine. We collected the supernatant using centrifugation for 10 min at 4 °C and 12 000 rpm and then used an enzyme‐linked immunosorbent assay (ELISA) to detect the cytokines.

##### In Vivo Antitumor Effects of PD‐L1‐GEM by Intravesical Instillation

An in situ bladder cancer model was created to determine PD‐L1‐GEMs’ in vivo antitumor activity. Ether inhalation was used to anesthetize female SD rats, followed by 22‐gage angiocatheter‐mediated infusion of 0.2 mL *N*‐methyl‐*N*‐nitrosourea (MNU) (10 mg mL^−1^; Sigma) into their bladders once every 14 d, 5 times. Postcatheterization, the rats were kept anesthetized for about 45 min to abrogate spontaneous micturition.

After the tumors were successfully induced, the rats anesthetized and placed into six groups (*n* = 10 per group) comprising PBS, PD‐L1, Control‐RS, GEM, Control‐RS‐GEMs, and PD‐L1‐GEMs (equivalent GEM = 5 mg kg^−1^) treatment via bladder infusion. Postinfusion, the rats were kept anesthetized for about 45 min to abrogate spontaneous micturition. The rats were treated once a week for five weeks. At two days after the last treatment, the rats were sacrificed humanely. Their excised bladders were fixed using 4% paraformaldehyde for 24 h, embedded in paraffin, and examined histopathologically. Transverse sections were cut from the midportion of the bladder and stained using hematoxylin and eosin (H&E).

##### Statistical Considerations

Statistical Product and Service Solutions SPSS 22.0 Software (IBM, Armonk, NY, USA) was used for all statistical analyses. Data were expressed as the mean ± standard deviation (X ± SD). The *χ*2 test was applied to examine the correlation between bladder cancer characteristics and PD‐L1 expression in bladder cancer cells. The Cox proportional hazard regression model was used to carry out univariate and multivariate analyses of prognosis. Kaplan–Meier curves were applied to assess survival and the differences between the curves were compared using the log‐rank test. Statistical significance was accepted if the *P* value was less than 0.05.

## Conflict of Interest

The authors declare no conflict of interest.

## Author Contributions

J.Y.L. and X.H. designed and performed experiments and data analyses and wrote the manuscript, J.Y.L., H.L.Z., Y.B.P., M.H.D., W.X., B.L., JH.L., Y.L.F., and Z.Q.H. performed data analysis, X.W.L., W.B.H., J.T., Z.L., and L.W. contributed to study design and manuscript editing.

## Supporting information

Supplementary Material

## Data Availability

The data that support the findings of this study are available in the supplementary material of this article.

## References

[1] A. T. Lenis , P. M. Lec , K. Chamie , M. D. Mshs , JAMA 2020, 324, 1980. 33201207 10.1001/jama.2020.17598

[2] G. Marcq , E. Jarry , I. Ouzaid , J. F. Hermieu , F. Henon , J. C. Fantoni , E. Xylinas , Ther. Adv. Urol. 2019, 11, 1756287218823678. 30728860 10.1177/1756287218823678 PMC6350113

[3] I. Rizzuto , E. Ghazaly , G. J. Peters , Pharmacogenomics 2017, 18, 911. 28594276 10.2217/pgs-2017-0034

[4] P. Gontero , G. Casetta , G. Maso , F. Sogni , G. Pretti , A. Zitella , B. Frea , A. Tizzani , Eur. Urol. 2004, 46, 339. 15306105 10.1016/j.eururo.2004.05.001

[5] R. Chou , S. Selph , D. I. Buckley , R. Fu , J. C. Griffin , S. Grusing , J. L. Gore , J. Urol. 2017, 197, 1189. 28027868 10.1016/j.juro.2016.12.090

[6] J. L. Godwin , J. Hoffman-Censits , E. Plimack , Urol. Oncol. 2018, 36, 109. 29395952 10.1016/j.urolonc.2017.12.018

[7] L. Tran , J. F. Xiao , N. Agarwal , J. E. Duex , D. Theodorescu , Nat. Rev. Cancer 2021, 21, 104. 33268841 10.1038/s41568-020-00313-1 PMC10112195

[8] P. Ardelt , A. Bohle , Eur. Urol. 2002, 41, 372; discussion 380-1. 12074806 10.1016/s0302-2838(02)00034-9

[9] S. Bagchi , R. Yuan , E. G. Engleman , Annu. Rev. Pathol.: Mech. Dis. 2021, 16, 223. 10.1146/annurev-pathol-042020-042741 33197221

[10] J. Gong , A. Chehrazi-Raffle , S. Reddi , R. Salgia , J. ImmunoTher. Cancer 2018, 6, 8. 29357948 10.1186/s40425-018-0316-z PMC5778665

[11] J. Hamanishi , M. Mandai , N. Matsumura , K. Abiko , T. Baba , I. Konishi , Int. J. Clin. Oncol. 2016, 21, 462. 26899259 10.1007/s10147-016-0959-z PMC4901122

[12] S. P. Patel , R. Kurzrock , Mol. Cancer Ther. 2015, 14, 847. 25695955 10.1158/1535-7163.MCT-14-0983

[13] L. Chen , X. Han , J. Clin. Invest 2015, 125, 3384. 26325035 10.1172/JCI80011 PMC4588282

[14] A. Lopez-Beltran , A. Cimadamore , A. Blanca , F. Massari , N. Vau , M. Scarpelli , L. Cheng , R. Montironi , Cancers 2021, 13, 131. 33401585 10.3390/cancers13010131 PMC7795541

[15] T. L. Rose , M. R. Harrison , A. M. Deal , S. Ramalingam , Y. E. Whang , B. Brower , M. Dunn , C. K. Osterman , H. M. Heiling , M. A. Bjurlin , A. B. Smith , M. E. Nielsen , H. J. Tan , E. Wallen , M. E. Woods , D. George , T. Zhang , A. Drier , W. Y. Kim , M. I. Milowsky , J. Clin. Oncol. 2021, 39, 3140. 34428076 10.1200/JCO.21.01003 PMC8478388

[16] J. Hu , J. Chen , Z. Ou , H. Chen , Z. Liu , M. Chen , R. Zhang , A. Yu , R. Cao , E. Zhang , X. Guo , B. Peng , D. Deng , C. Cheng , J. Liu , H. Li , Y. Zou , R. Deng , G. Qin , W. Li , L. Wang , T. Chen , X. Pei , G. Gong , J. Tang , B. Othmane , Z. Cai , C. Zhang , Z. Liu , X. Zu , Cell Rep. Med. 2022, 3, 100785. 36265483 10.1016/j.xcrm.2022.100785 PMC9729796

[17] X. Liu , J. Jiang , Y. P. Liao , I. Tang , E. Zheng , W. Qiu , M. Lin , X. Wang , Y. Ji , K. C. Mei , Q. Liu , C. H. Chang , Z. A. Wainberg , A. E. Nel , H. Meng , Adv. Sci. 2021, 8, 2002147. 10.1002/advs.202002147 PMC7967046 33747719

[18] W. Xiang , Y. Peng , H. Zeng , C. Yu , Q. Zhang , B. Liu , J. Liu , X. Hu , W. Wei , M. Deng , N. Wang , X. Liu , J. Xie , W. Hou , J. Tang , Z. Long , L. Wang , J. Liu , Biomater. Res. 2022, 26, 74. 36471380 10.1186/s40824-022-00328-9 PMC9721011

[19] M. Chen , Y. Yu , F. Jiang , J. Zhou , Y. Li , C. Liang , L. Dang , A. Lu , G. Zhang , Int. J. Mol. Sci. 2016, 17, 2079. 27973403 10.3390/ijms17122079 PMC5187879

[20] J. Canoura , H. Yu , O. Alkhamis , D. Roncancio , R. Farhana , Y. Xiao , J. Am. Chem. Soc. 2021, 143, 805. 33378616 10.1021/jacs.0c09559 PMC7855447

[21] J. Kim , W. Park , D. Kim , E. S. Lee , D. H. Lee , S. Jeong , J. M. Park , K. Na , Adv. Funct. Mater. 2019, 29, 1900084.

[22] S. M. Nimjee , R. R. White , R. C. Becker , B. A. Sullenger , Annu. Rev. Pharmacol. Toxicol. 2017, 57, 61. 28061688 10.1146/annurev-pharmtox-010716-104558 PMC6035745

[23] N. Li , C. M. Ho , J. Am. Chem. Soc. 2008, 130, 2380. 18247609 10.1021/ja076787b

[24] F. Radom , P. M. Jurek , M. P. Mazurek , J. Otlewski , F. Jelen , Biotechnol. Adv. 2013, 31, 1260. 23632375 10.1016/j.biotechadv.2013.04.007

[25] G. Zhu , X. Chen , Adv. Drug Delivery Rev. 2018, 134, 65. 10.1016/j.addr.2018.08.005 PMC6239901 30125604

[26] S. M. Hammond , A. Aartsma-Rus , S. Alves , S. E. Borgos , R. A. M. Buijsen , R. W. J. Collin , G. Covello , M. A. Denti , L. R. Desviat , L. Echevarria , C. Foged , G. Gaina , A. Garanto , A. T. Goyenvalle , M. Guzowska , I. Holodnuka , D. R. Jones , S. Krause , T. Lehto , M. Montolio , W. Van Roon-Mom , V. Arechavala-Gomeza , EMBO Mol. Med. 2021, 13, e13243. 10.15252/emmm.202013243 PMC8033518 33821570

[27] R. L. Letsinger , W. B. Lunsford , J. Am. Chem. Soc. 1976, 98, 3655. 1270704 10.1021/ja00428a045

[28] G. Zhu , G. Niu , X. Chen , Bioconjugate Chem. 2015, 26, 2186. 10.1021/acs.bioconjchem.5b00291 PMC5244258 26083153

[29] W. Y. Lai , B. T. Huang , J. W. Wang , P. Y. Lin , P. C. Yang , Mol. Ther. Nucleic Acids 2016, 5, e397. 10.1038/mtna.2016.102 27959341

[30] A. Zheng , Y. Du , Y. Wang , Y. Zheng , Z. Ning , M. Wu , C. Zhang , D. Zhang , J. Liu , X. Liu , Mol. Ther. Nucleic Acids 2022, 27, 998. 35228895 10.1016/j.omtn.2022.01.010 PMC8844804

[31] M. Passariello , S. Camorani , C. Vetrei , L. Cerchia , C. De Lorenzo , Cancers 2019, 11, 1268. 31470510 10.3390/cancers11091268 PMC6770524

[32] Y. Du , D. Zhang , Y. Wang , M. Wu , C. Zhang , Y. Zheng , A. Zheng , X. Liu , Biomater. Sci. 2021, 9, 4159. 33970170 10.1039/d0bm02210a

[33] M. A. Han , P. Maisch , J. H. Jung , J. E. Hwang , V. Narayan , A. Cleves , E. C. Hwang , P. Dahm , Cochrane Database Syst. Rev. 2021, 6, CD009294. 34125951 10.1002/14651858.CD009294.pub3 PMC8202966

[34] Z. Payandeh , S. Khalili , M. H. Somi , M. Mard-Soltani , A. Baghbanzadeh , K. Hajiasgharzadeh , N. Samadi , B. Baradaran , J. Cell Physiol. 2020, 235, 5461. 31960962 10.1002/jcp.29494

[35] H. Yu , T. A. Boyle , C. Zhou , D. L. Rimm , F. R. Hirsch , J. Thorac. Oncol. 2016, 11, 964. 27117833 10.1016/j.jtho.2016.04.014 PMC5353357

[36] F. L. Ricklefs , Q. Alayo , H. Krenzlin , A. B. Mahmoud , M. C. Speranza , H. Nakashima , J. L. Hayes , K. Lee , L. Balaj , C. Passaro , A. K. Rooj , S. Krasemann , B. S. Carter , C. C. Chen , T. Steed , J. Treiber , S. Rodig , K. Yang , I. Nakano , H. Lee , R. Weissleder , X. O. Breakefield , J. Godlewski , M. Westphal , K. Lamszus , G. J. Freeman , A. Bronisz , S. E. Lawler , E. A. Chiocca , Sci. Adv. 2018, 4, eaar2766. 10.1126/sciadv.aar2766 PMC5842038 29532035

[37] G. Qin , X. Wang , S. Ye , Y. Li , M. Chen , S. Wang , T. Qin , C. Zhang , Y. Li , Q. Long , H. Hu , D. Shi , J. Li , K. Zhang , Q. Zhai , Y. Tang , T. Kang , P. Lan , F. Xie , J. Lu , W. Deng , Nat. Commun. 2020, 11, 1669. 32245950 10.1038/s41467-020-15364-z PMC7125142

[38] F. Li , J. Lu , J. Liu , C. Liang , M. Wang , L. Wang , D. Li , H. Yao , Q. Zhang , J. Wen , Z. K. Zhang , J. Li , Q. Lv , X. He , B. Guo , D. Guan , Y. Yu , L. Dang , X. Wu , Y. Li , G. Chen , F. Jiang , S. Sun , B. T. Zhang , A. Lu , G. Zhang , Nat. Commun. 2017, 8, 1390. 29123088 10.1038/s41467-017-01565-6 PMC5680242

[39] S. Yan , H. Zeng , K. Jin , F. Shao , Z. Liu , Y. Chang , Y. Wang , Y. Zhu , Z. Wang , L. Xu , J. Xu , J. ImmunoTher. Cancer 2022, 10, e004569. 10.1136/jitc-2022-004569 PMC9073407 35523436

[40] J. H. Cha , L. C. Chan , C. W. Li , J. L. Hsu , M. C. Hung , Mol. Cell 2019, 76, 359. 31668929 10.1016/j.molcel.2019.09.030 PMC6981282

[41] D. F. Bajorin , J. A. Witjes , J. E. Gschwend , M. Schenker , B. P. Valderrama , Y. Tomita , A. Bamias , T. Lebret , S. F. Shariat , S. H. Park , D. Ye , M. Agerbaek , D. Enting , R. McDermott , P. Gajate , A. Peer , M. I. Milowsky , A. Nosov , J. K. Neif AntonioTupikowski , Jr Tupikowski , K. , L. Toms , B. S. Fischer , A. Qureshi , S. Collette , K. Unsal-Kacmaz , E. Broughton , D. Zardavas , H. B. Koon , M. D. Galsky , N. Engl. J. Med. 2021, 384, 2102. 34077643 10.1056/NEJMoa2034442 PMC8215888

[42] B. A. Inman , T. A. Longo , S. Ramalingam , M. R. Harrison , Clin. Cancer Res. 2017, 23, 1886. 27903674 10.1158/1078-0432.CCR-16-1417

[43] International Collaboration of Trialists , Medical Research Council Advanced Bladder Cancer Working Party , European Organisation for Research and Treatment of Cancer Genito‐Urinary Tract Cancer Group , Australian Bladder Cancer Study Group , National Cancer Institute of Canada Clinical Trials Group , Finnbladder , Norwegian Bladder Cancer Study Group , Club Urologico Espanol de Tratamiento Oncologico Group , G. Griffiths , R. Hall , R. Sylvester , D. Raghavan , M. K. Parmar , J. Clin. Oncol. 2011, 29, 2171.21502557

[44] H. D. Patel , S. H. Patel , E. Blanco-Martinez , J. Kuzbel , V. S. Chen , A. Druck , E. L. Koehne , P. M. Patel , C. P. Doshi , N. M. Hahn , J. H. Hoffman-Censits , S. Berg , T. J. Bivalacqua , M. Kates , M. L. Quek , J. Urol. 2022, 207, 77. 34445890 10.1097/JU.0000000000002189

[45] D. D’Andrea , P. C. Black , H. Zargar , C. P. Dinney , F. Soria , M. S. Cookson , J. S. Montgomery , W. Kassouf , M. A. Dall’Era , S. S. Sridhar , J. S. McGrath , J. L. Wright , A. C. Thorpe , J. M. Holzbeierlein , D. M. Carrion , E. Di Trapani , T. J. Bivalacqua , S. North , D. A. Barocas , Y. Lotan , P. Grivas , A. J. Stephenson , B. W. van Rhijn , S. Daneshmand , P. E. Spiess , S. F. Shariat , Contributors , J. Urol. 2022, 207, 70. 34445891 10.1097/JU.0000000000002190

[46] J. Xu , Y. Bai , N. Xu , E. Li , B. Wang , J. Wang , X. Li , X. Wang , X. Yuan , Clin. Cancer Res. 2020, 26, 4542. 32561664 10.1158/1078-0432.CCR-19-3561

[47] J. Wang , S. Lu , X. Yu , Y. Hu , Y. Sun , Z. Wang , J. Zhao , Y. Yu , C. Hu , K. Yang , G. Feng , K. Ying , W. Zhuang , J. Zhou , J. Wu , S. J. Leaw , J. Zhang , X. Lin , L. Liang , N. Yang , JAMA Oncol. 2021, 7, 709. 33792623 10.1001/jamaoncol.2021.0366 PMC8017481

[48] S. Lu , J. Wang , Y. Yu , X. Yu , Y. Hu , X. Ai , Z. Ma , X. Li , W. Zhuang , Y. Liu , W. Li , J. Cui , D. Wang , W. Liao , J. Zhou , Z. Wang , Y. Sun , X. Qiu , J. Gao , Y. Bao , L. Liang , M. Wang , J. Thorac. Oncol. 2021, 16, 1512. 34033975 10.1016/j.jtho.2021.05.005

[49] J. Y. Liu , Y. B. Dai , X. Li , K. Cao , D. Xie , Z. T. Tong , Z. Long , H. Xiao , M. K. Chen , Y. L. Ye , B. Liu , J. Tan , J. Tang , Z. Z. Xu , Y. Gan , Y. H. Zhou , F. Deng , L. Y. He , Cell Death Dis. 2017, 8, e2691. 10.1038/cddis.2017.118 PMC5386524 28333147

[50] B. Wang , W. Pan , M. Yang , W. Yang , W. He , X. Chen , J. Bi , N. Jiang , J. Huang , T. Lin , Cancer Sci. 2019, 110, 489. 30548363 10.1111/cas.13887 PMC6361576

[51] R. S. Herbst , J. C. Soria , M. Kowanetz , G. D. Fine , O. Hamid , M. S. Gordon , J. A. Sosman , D. F. McDermott , J. D. Powderly , S. N. Gettinger , H. E. Kohrt , L. Horn , D. P. Lawrence , S. Rost , M. Leabman , Y. Xiao , A. Mokatrin , H. Koeppen , P. S. Hegde , I. Mellman , D. S. Chen , F. S. Hodi , Nature 2014, 515, 563. 25428504 10.1038/nature14011 PMC4836193

