# Supplementary material for: Targeting PD‐L1 with DNA Aptamers and Conjugated with Gemcitabine as a Novel Therapeutic Strategy for Bladder Cancer Chemotherapy Combined with Immunotherapy
Source: Small Sci. 2023 Oct 17;3(12):2300104. doi: 10.1002/smsc.202300104 (PMC11936014; doi:10.1002/smsc.202300104)
Supplement: Supplementary file 1 — Supplementary Material [file SMSC-3-2300104-s001.pdf]

Supporting Information

Targeting PD-L1 with DNA aptamers and conjugated with gemcitabine as a therapeutic strategy for bladder cancer chemotherapy combined with immunotherapy

Xing Hu, Hongliang Zeng, Yongbo Peng, Minhua Deng, Wei Xiang, Biao Liu, Jiahao Liu, Yunlong Fu, Zhiqiang Hu, Weibin Hou, Xuewen Liu, Jin Tang, Zhi Long, Long Wang, and Jianye Liu*

Oligonucleotide synthesis and purification

As shown in **Figure S1**[1], 1350 mg of Lutidine and 900 mg of Li_2CO_3, suspended in 100 mL of anhydrous CH_2Cl_2, were added to compound 1 (269 mg of gemcitabine, approximately 1.0 mmol, 97% pure). Next, we added 460 mg (1.12. mol) of 4,4'-dimethoxytrityl tetrafluoroborate salt ($DMTBF_4$) and 150 mL of CH_2Cl_2, and the precipitate was washed using saturated NaCl and dried in the presence of anhydrous Na_2SO_4. The solvent was removed, and a flash column was used to purify the residue to provide compound 2(approximately 400 mg, $M+H^+$ = 543.3, yield = 73%). Then, CH_2Cl_2 was used to resuspend 246 mg (approximately 0.5mmol) of compound 2, 650 mg (5 mmol) of N, N-Diisopropylethylamine (DIEA) was added, and the mixture was cooled at 0 ℃ Next, we added 596 g (2.44 mmol)) of N-diisopropylchlorophosphoramidite and then monitored the reaction using thin-layer chromatography (TLC). When all the starting material had dissolved, CH_2Cl_2 (~100 mL) was used to dilute the reaction solution, followed by washing with saturated NaCl and saturated

$NaHCO_3$. The products were dried over anhydrous Na_2SO_4, concentrated, and a flash column was used to purify the residue, giving approximately 380 mg of GEM phosphoramidite 3 as a white powder at a yield of 80% (MW calculated for 950.03; M+Na$^+$ = 973.09). Compared with the calculated structure, the MS, ^{1}H-NMR, and ^{13}C-NMR of the product were consistent (**Figure. S2-S4**)[2].

After the synthesis of the PD-L1-GEMs sequence, a DNA synthesizer (PolyGen GmbH, Langen, Germany) was used to carry out the next procedure in auto-synthesis mode, following the supplier's protocol. Previously reported standards were used to treat the primary products. To cleave the CpG of dinucleotides, the primary products were reacted with approximately 400 µl of 28% ammonium hydroxide for 30 min at 65 ℃ One ml of ice-cold ethanol together with 40 µL of 3 M NaCl were mixed with the cleaved DNA, followed by incubation —20 ℃for 1 h. The reaction was then centrifuged at 12,000 rpm and 4 ℃for 20 min and the supernatant was discarded. The pellet was suspended in 1 mL of ice-cold ethanol and 40 µL of 3 M NaCl, left to stand for 1 h at —20 ℃to allow the oligonucleotides to precipitate, followed by repeated centrifugation to obtain the pellet. Triethylamine acetate (TEAA; 400 µL, 0.1 M) was used to dissolve the pellet, which was then purified using HPLC. After lyophilization, the oligonucleotides were dissolved using sterilized ultrapure water and subjected to desalting via a desalt mini column. Next, fluorescein isothiocyanate (FITC) and cyanine 5 (cy5) labeling of the strands was carried out, followed by further purification. After quantification, the oligonucleotides in sterilized water were stored for subsequent experiments.

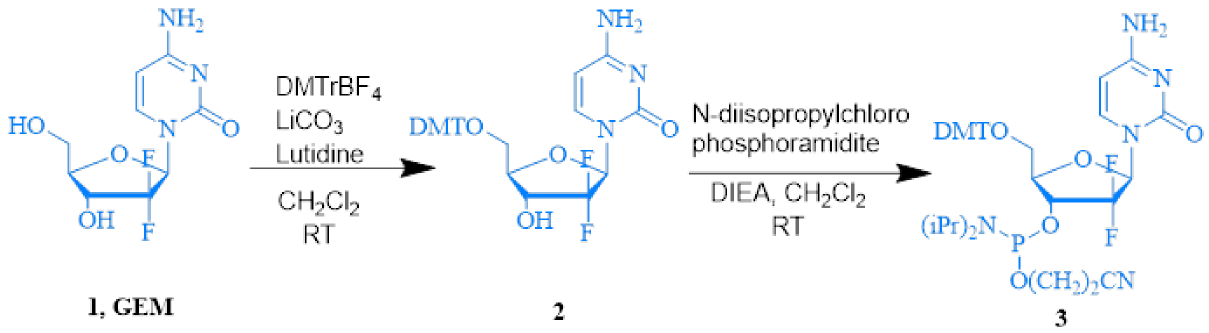

Figure S1. The synthesis of GEM phosphoramidite 3.

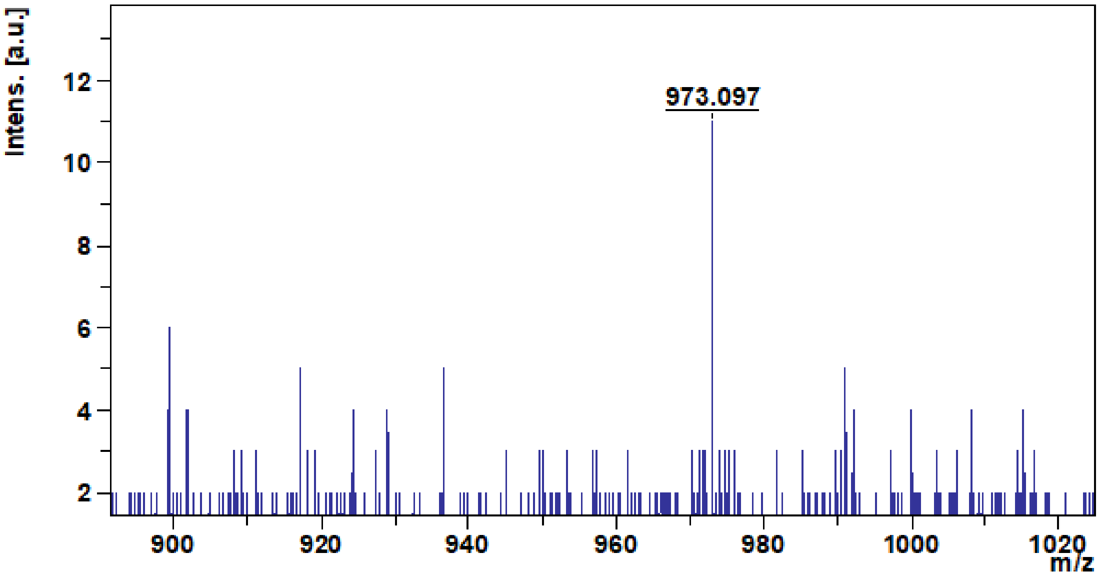

Figure S2. The mass spectrum of GEM phosphoramidite 3.

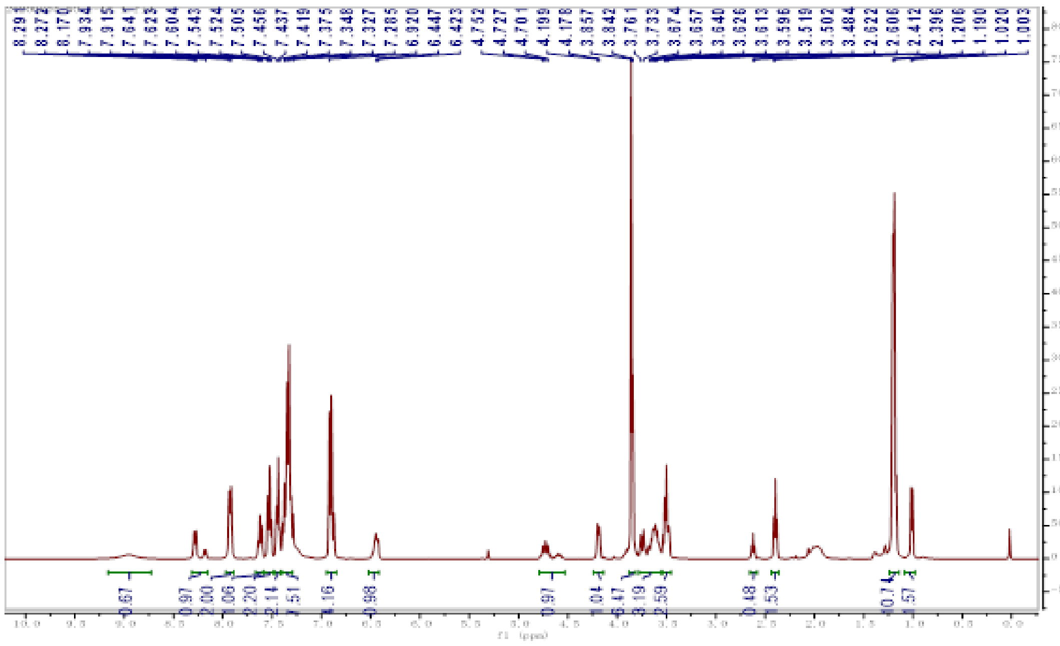

Figure S3. The ^{1}H-NMR spectrum of GEM phosphoramidite 3.

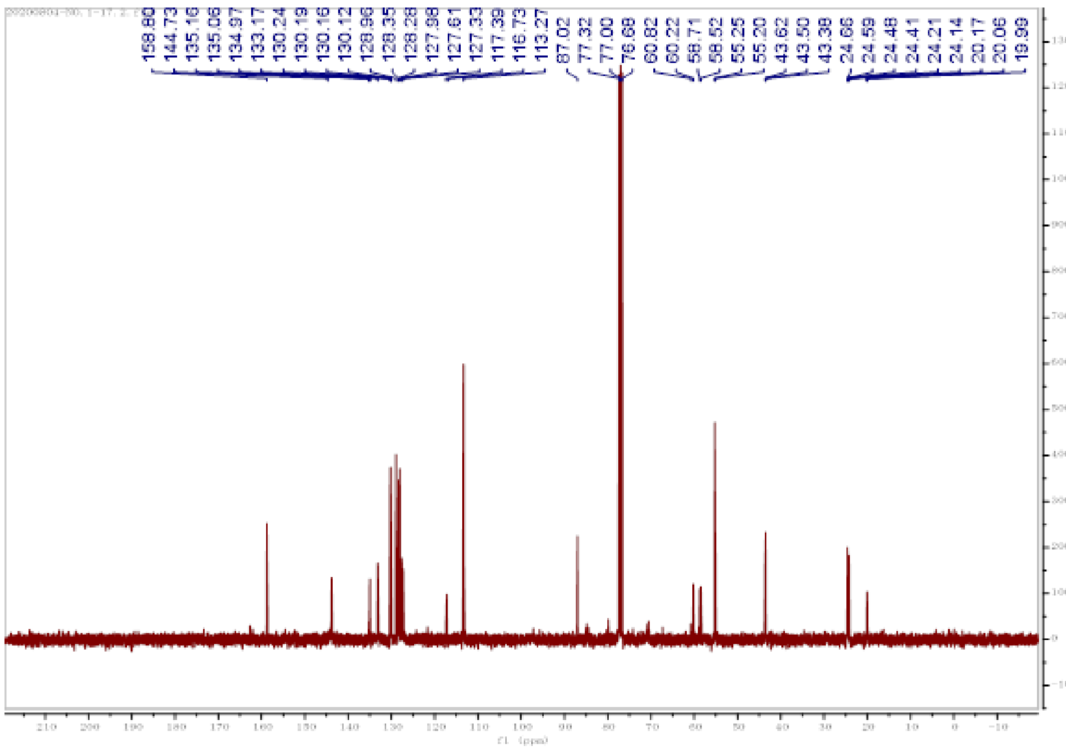

Figure S4. The ^{13}C-NMR spectrum of GEM phosphoramidite 3.

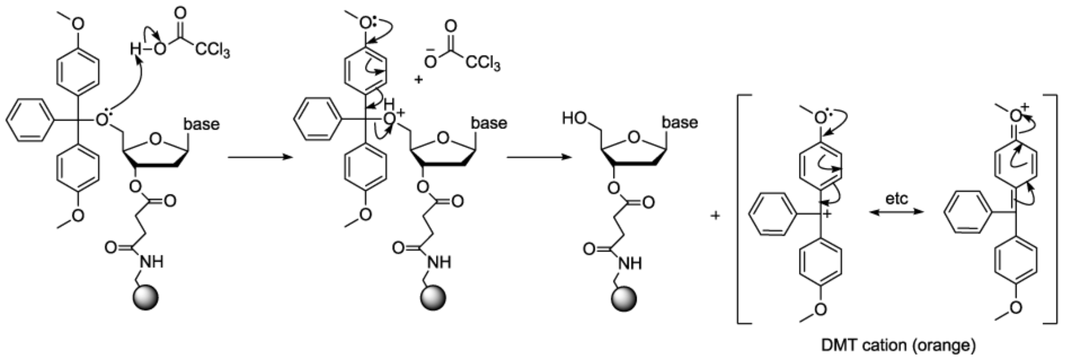

Figure S5. Step one of synthesis: According to the 3 'terminal first base of the oligonucleotide, CPG linked with one of the bases A, G, C and T is selected as the solid carrier. The base 5' OH is protected by the DMT protection group. Before starting synthesis, remove the DMT of the first base with trichloroacetic acid, and expose the active 5 'OH.

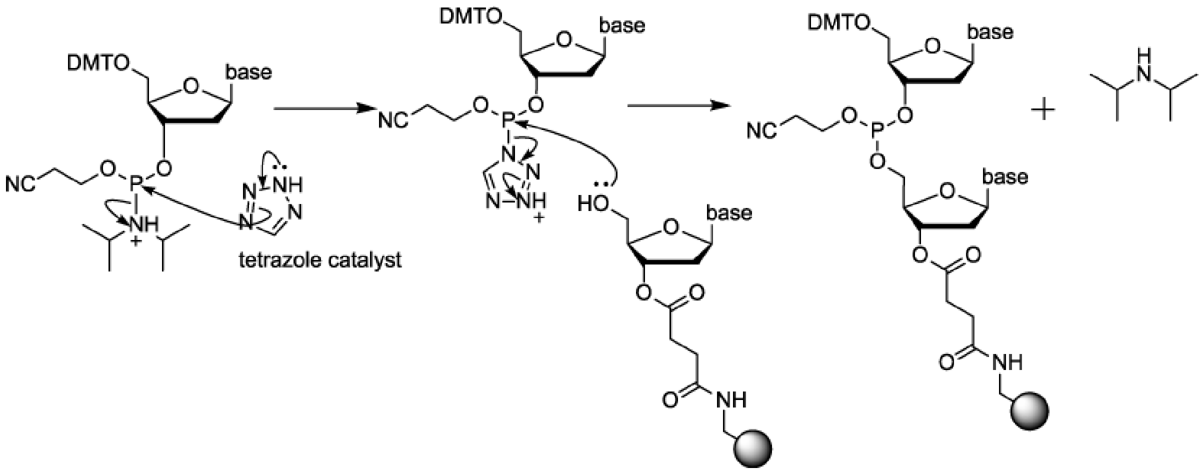

Figure S6. Coupling: After the completion of DMT removal, clean the residual trichloroacetic acid on the solid carrier with anhydrous acetonitrile, add tetrazolium and corresponding phosphorous amide monomer, and the phosphorous amide monomer activated by tetrazolium reacts with 5 'OH on the solid carrier to form phosphorous ester bond.

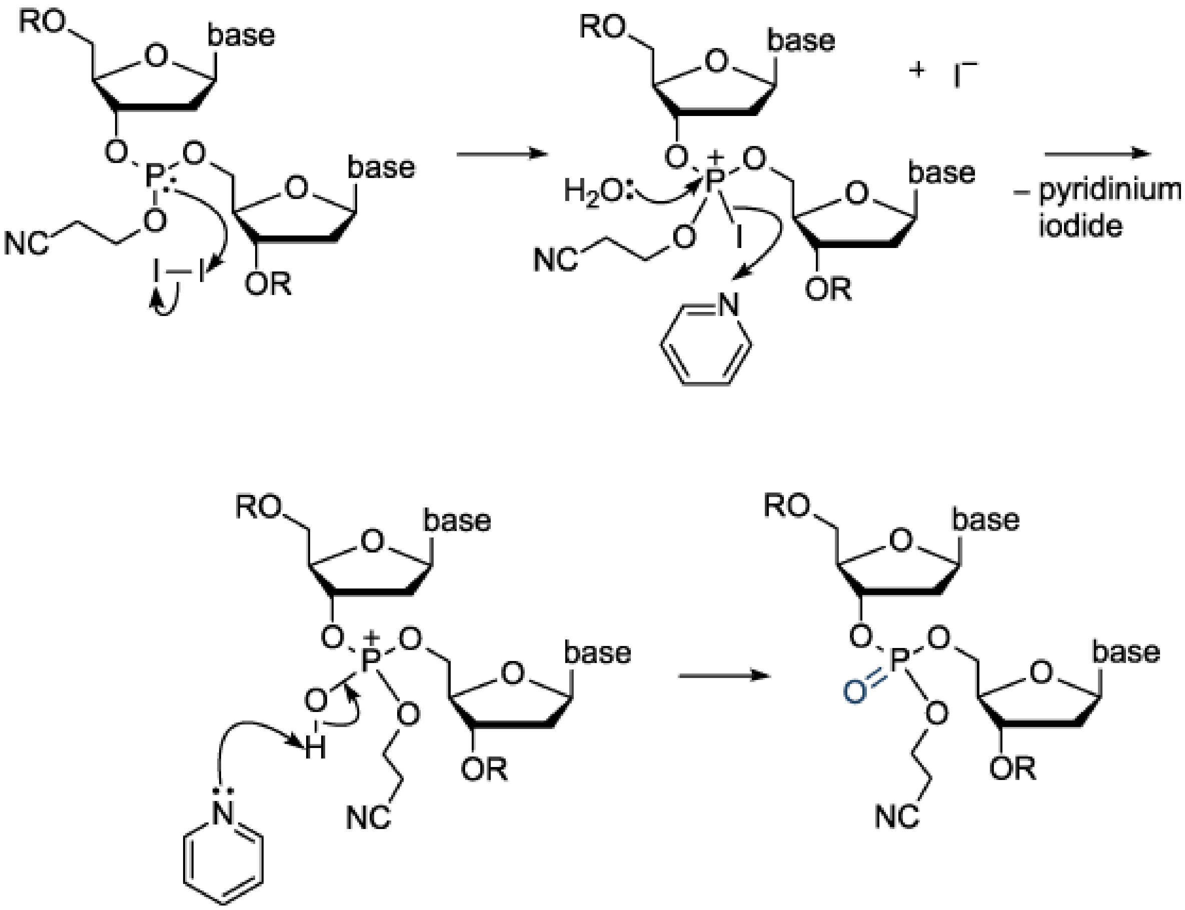

Figure S7. Oxidation: The phosphorous ester bond formed in the coupling step is unstable under acidic conditions. It is necessary to use I2 to oxidize trivalent phosphorus to pentavalent phosphorus to form a stable phosphate diester bond.

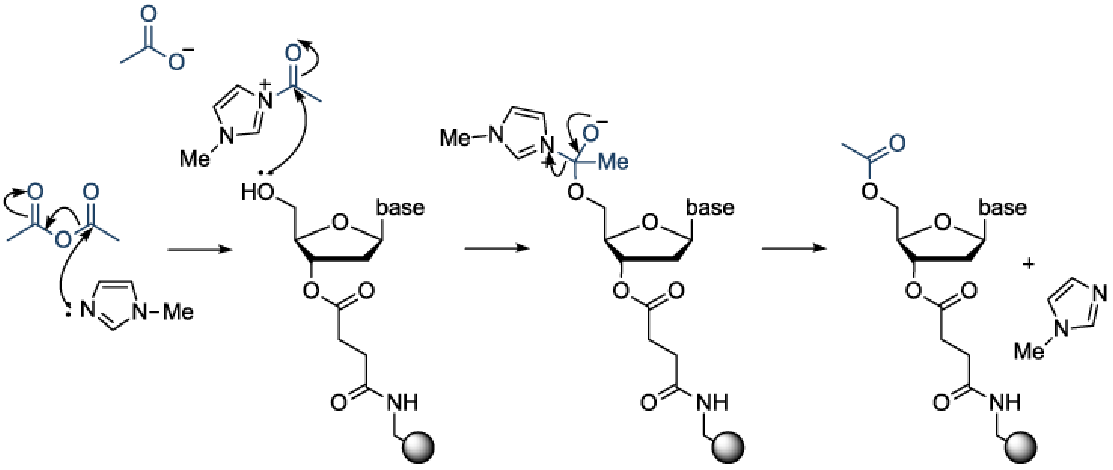

Figure S8. Capping: Use acetyl group to block 5 'OH that is not involved in the reaction to avoid its participation in the next round of reaction.

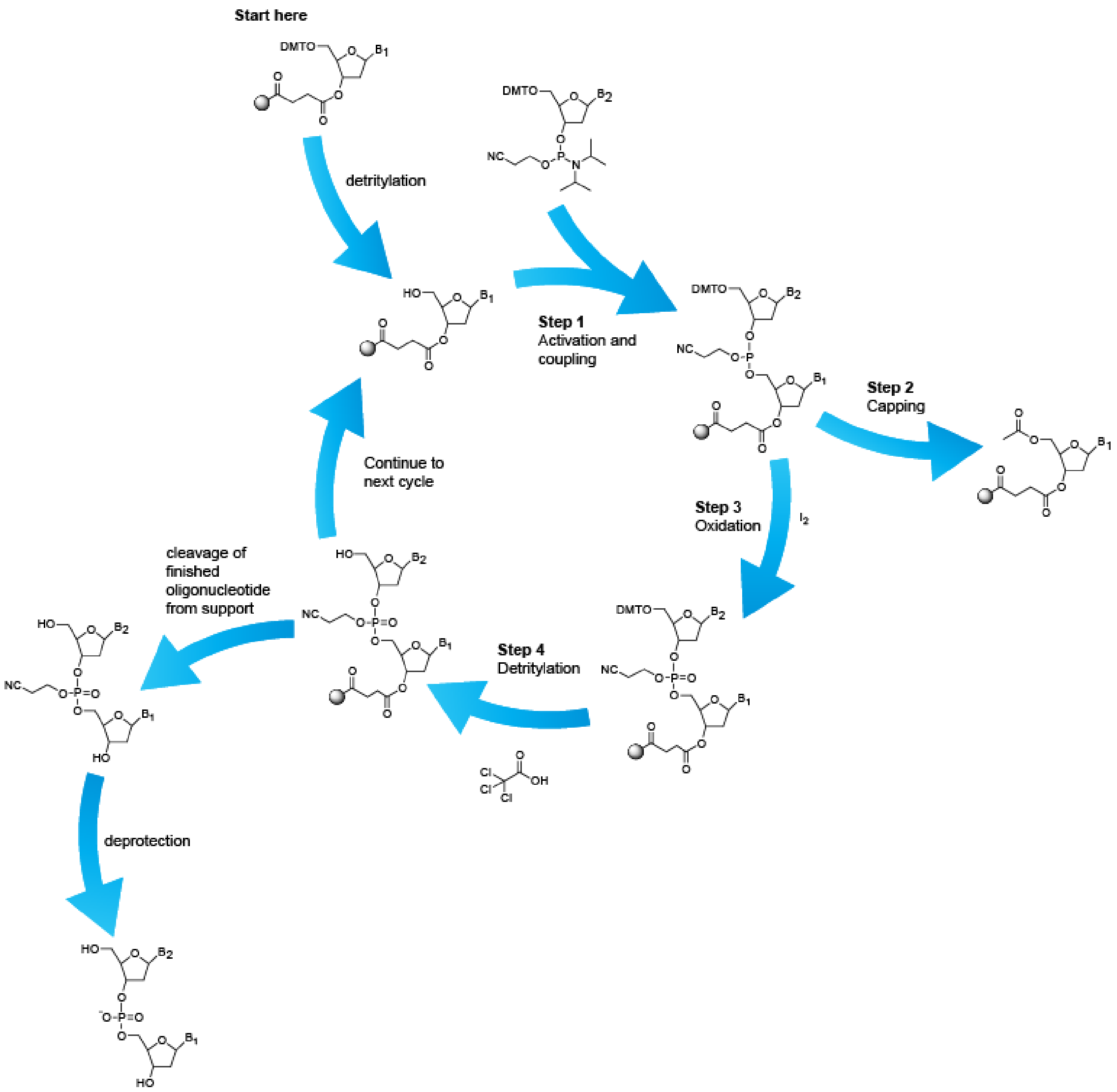

Figure S9. The synthesis of PD-L1-GEMs.

**Name :**	**PD-L1-Aptamer**		
**Sequence(5'to3'):**	TTTACAGGTTCTGGGGGGTGGGTGGGGAACCTGTTTT		
**Lot No. :**	AX204071617	**Length :**	37
**Purification :**	HPLC	**Modification(5'to3):**	
**nmoles:**	5.72	**Add water to 100uM:**	57.2
**TM(℃) :**	71	**GC(%) :**	54.1
**MW (target):**	11569.48	**MW(observed):**	11566.8
**Conclusion:**		**Qualified:**	
**Inspector:**		**Auditor:**	

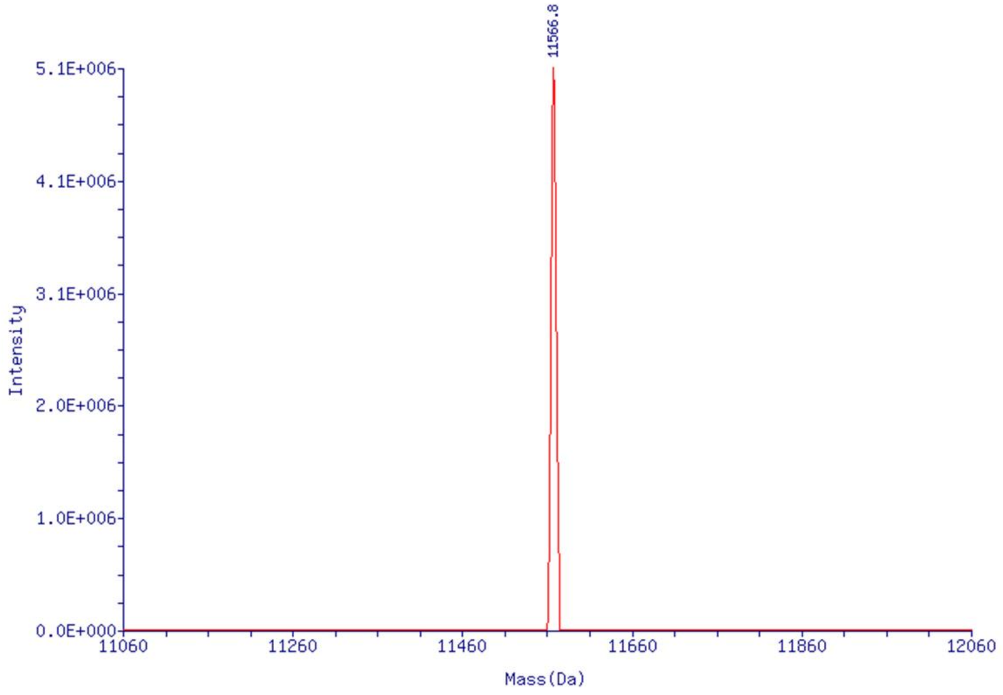

Figure S10. ESI-MS spectrum of PD-L1 aptamer.

**Name :**	**Control-RS**		
**Sequence(5'to3'):**	TTATTACCTCTAAATCACTGCTCTGTAACATGGTCGCGCTAGGTT		
**Lot No. :**	AX204071616	**Length :**	45
**Purification :**	HPLC	**Modification(5'to3'):**	
**nmoles:**	4.76	**Add water to 100uM:**	47.6
**TM(℃) :**	68.5	**GC(%) :**	42.2
**MW (target):**	13751.95	**MW(observed):**	13749.3
**Conclusion:**		**Qualified:**	
**Inspector:**		**Auditor:**	

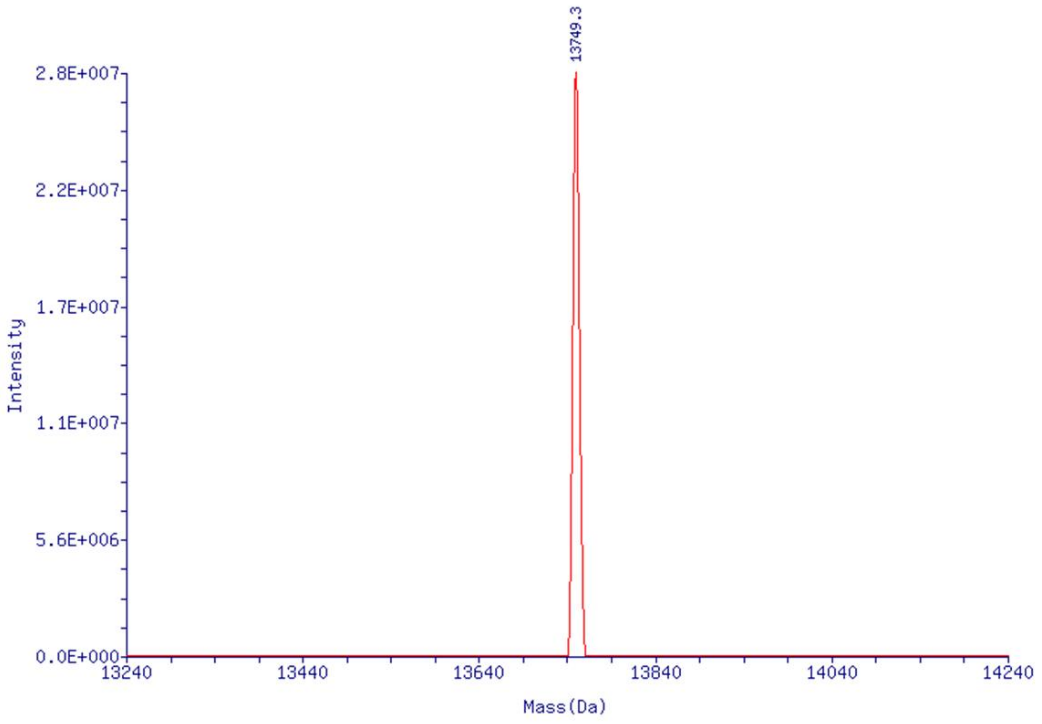

Figure S11. ESI-MS spectrum of Control-RS.

**Name ：**	**PD-L1 Aptamer-FITC**		
**Sequence(5'to3'):**	TTTACAGGTTCTGGGGGGTGGGTGGGGAACCTGTTTT		
**Lot No. :**	AX204071613	**Length :**	37
**Purification :**	HPLC	**Modification(5'to3'):**	3`FITC
**nmoles:**	5.4	**Add water to 100uM:**	54
**TM(℃) :**	71	**GC(%) :**	54.1
**MW (target):**	12140.55	**MW(observed):**	12136.2
**Conclusion:**		**Qualified:**	
**Inspector:**		**Auditor:**	

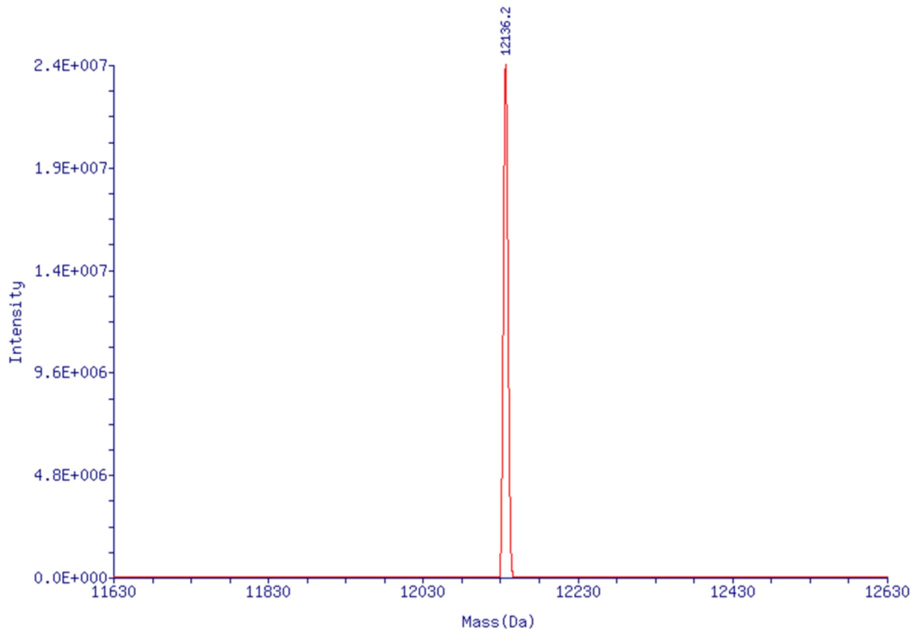

Figure S12. ESI-MS spectrum of PD-L1-FITC.

**Name :**	**Control-RS-FITC**		
**Sequence(5'to3'):**	TTATTACCTCTAAATCACTGCTCTGTAACATGGTCGCGCTAGGTT		
**Lot No. :**	AX204071612	**Length :**	45
**Purification :**	HPLC	**Modification(5'to3'):**	3`FITC
**nmoles:**	4.54	**Add water to 100uM:**	45.4
**TM(℃) :**	68.5	**GC(%) :**	42.2
**MW (target):**	14323.02	**MW(observed):**	14317.7
**Conclusion:**		**Qualified:**	
**Inspector:**		**Auditor:**	

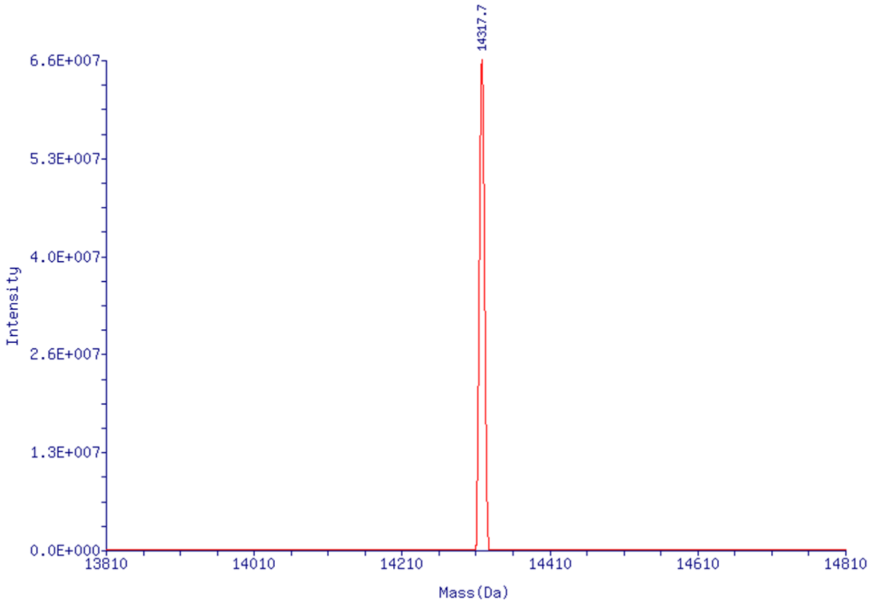

Figure S13. ESI-MS spectrum of Control-RS-FITC.

**Name :**	**PD-L1 Aptamer-cy5**		
**Sequence(5'to3'):**	TTTACAGGTTCTGGGGGGTGGGTGGGGAACCTGTTTT		
**Lot No. :**	AX209143498	**Length :**	37
**Purification :**	HPLC	**Modification(5'to3'):**	3`Cy5
**nmoles:**	5.56	**Add water to 100uM:**	55.6
**TM(℃) :**	71	**GC(%) :**	54.1
**MW (target):**	12102.48	**MW(observed):**	12101.6
**Conclusion:**		**Qualified:**	
**Inspector:**		**Auditor:**	

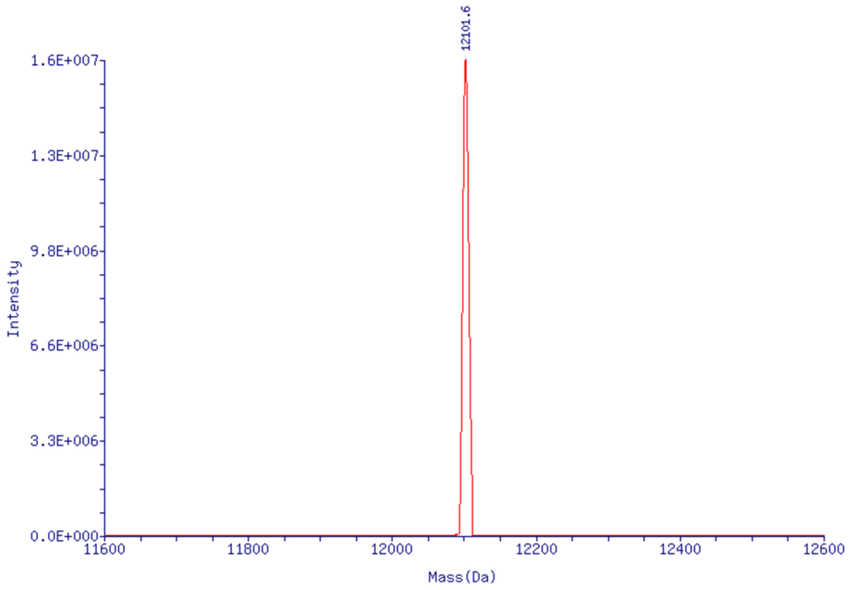

Figure S14. ESI-MS spectrum of PD-L1-cy5

**Name :**	**Control-RS-cy5**		
**Sequence(5'to3'):**	TTATTACCTCTAAATCACTGCTCTGTAACATGGTCGCGCTAGGTT		
**Lot No. :**	AX209143497	**Length :**	45
**Purification :**	HPLC	**Modification(5'to3'):**	3`Cy5
**nmoles:**	4.66	**Add water to 100uM:**	46.6
**TM(℃) :**	68.5	**GC(%) :**	42.2
**MW (target):**	14284.95	**MW(observed):**	14283.1
**Conclusion:**		**Qualified:**	
**Inspector:**		**Auditor:**	

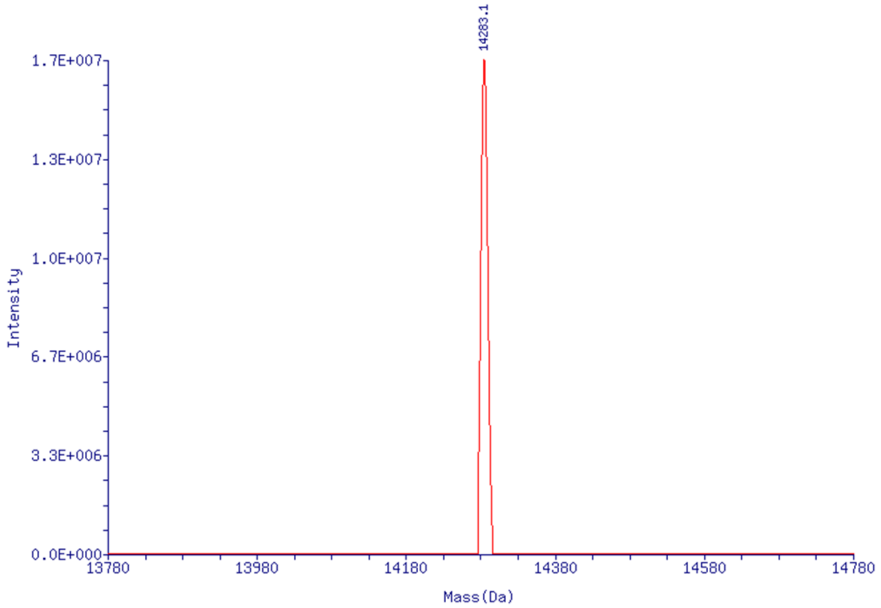

Figure S15. ESI-MS spectrum of Control-RS-cy5.

**Name :**	**PD-L1 Aptamer-3GEM**		
**Sequence(5'to3'):**	TTTACAGGTTCTGGGGGGTGGGTGGGGAACCTGTT/iGem//iGem//iGem/TT		
**Lot No. :**	AX204071615	**Length :**	40
**Purification :**	HPLC	**Modification(5'to3'):**	3Gem
**nmoles:**	50	**Add water to 100uM:**	500
**TM(℃) :**	73.4	**GC(%) :**	57.5
**MW (target):**	12544.62	**MW(observed):**	12541.8
**Conclusion:**		**Qualified:**	
**Inspector:**		**Auditor:**	

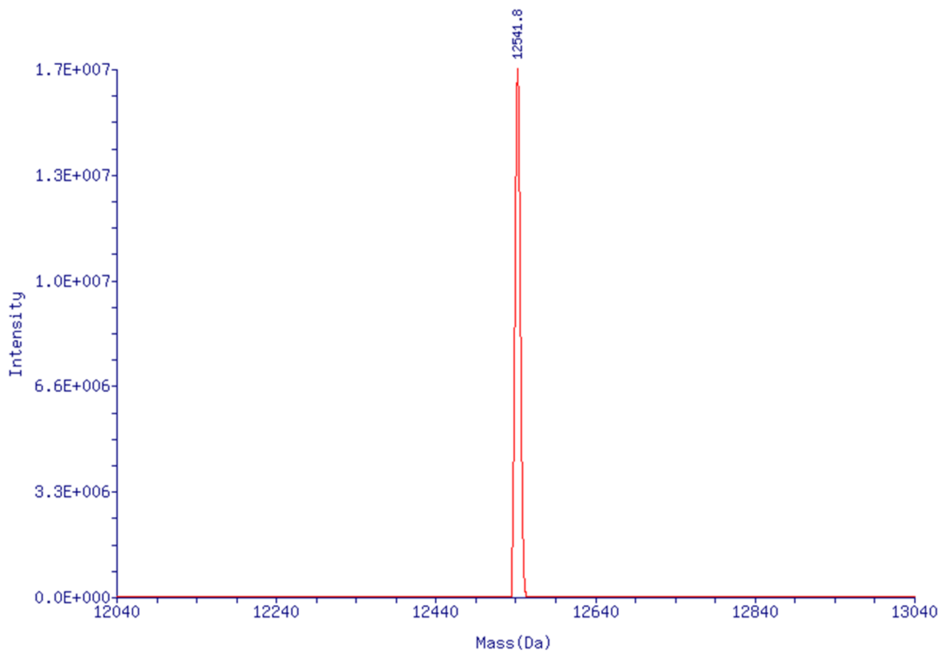

Figure S16. ESI-MS spectrum of PD-L1-GEMs.

**Name ：**	**Control-RS-3GEM**		
**Sequence(5'to3'):**	TTATTACCTCTAAATCACTGCTCTGTAACATGGTCGCGCTAGG/iGem//iGem//iGem/TT		
**Lot No. :**	AX204071614	**Length :**	48
**Purification :**	HPLC	**Modification(5'to3'):**	3Gem
**nmoles:**	50	**Add water to 100uM:**	500
**TM(℃) :**	70.7	**GC(%) :**	45.8
**MW (target):**	14727.09	**MW(observed):**	14724.0
**Conclusion:**		**Qualified:**	
**Inspector:**		**Auditor:**	

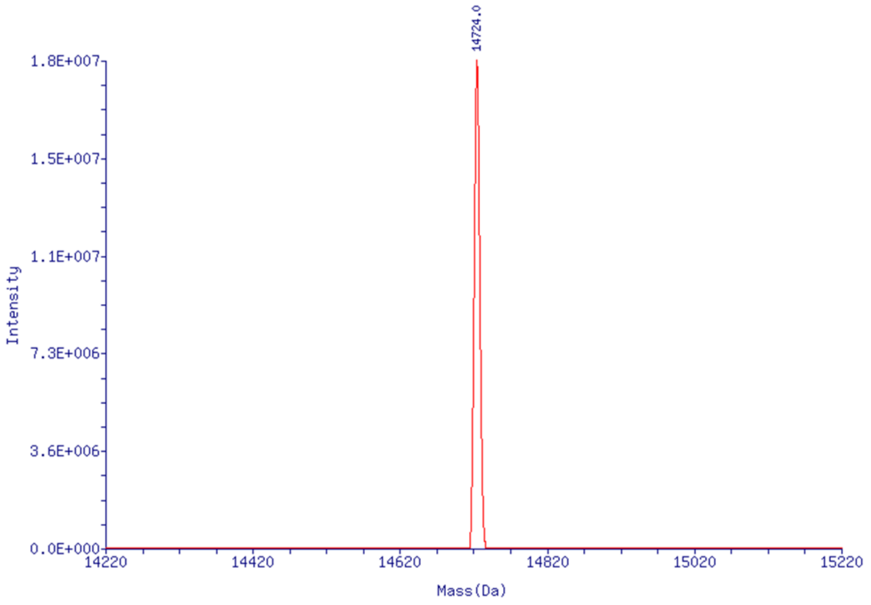

Figure S17. ESI-MS spectrum of Control-RS-GEMs.

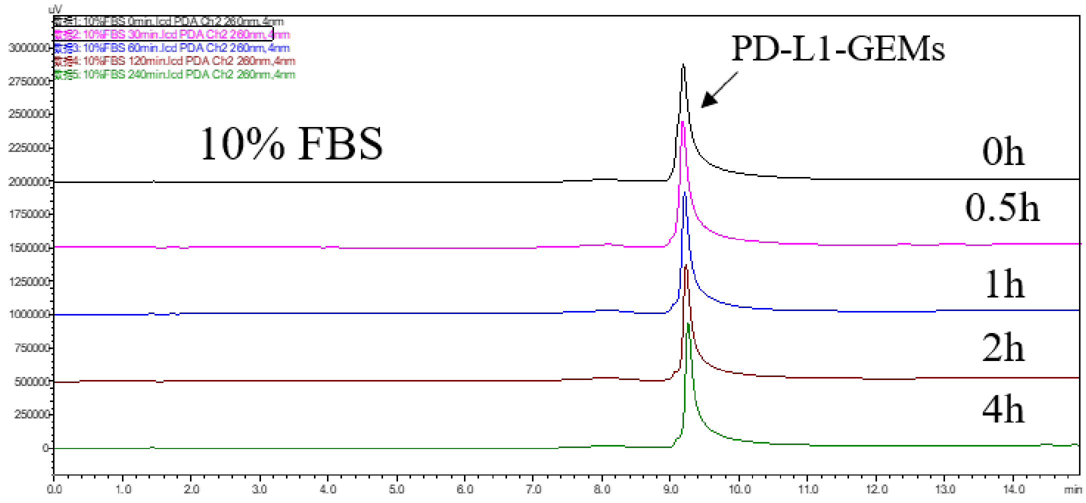

Figure S18. The release of GEM from PD-L1-GEMs in 10% FBS buffer.

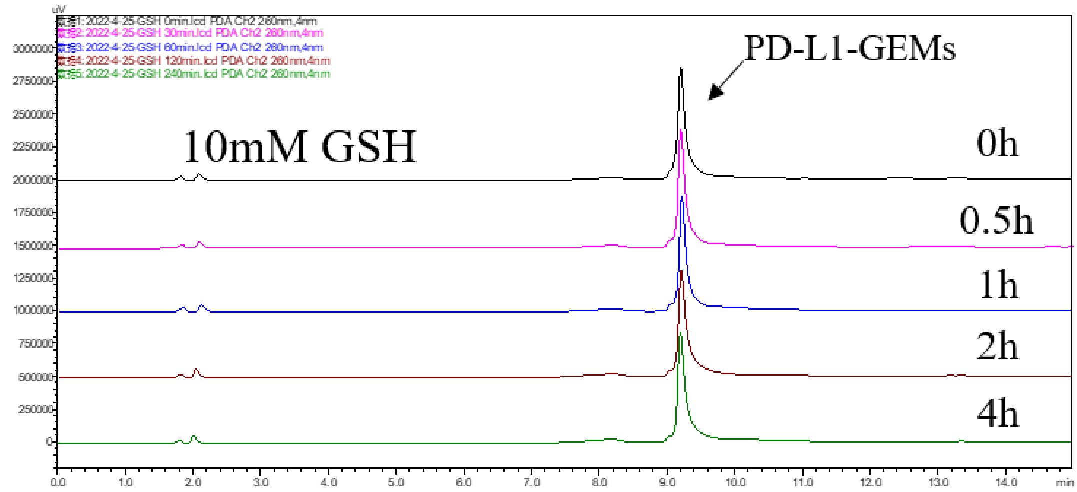

Figure S19. The release of GEM from PD-L1-GEMs in 10 mM GSH buffer.

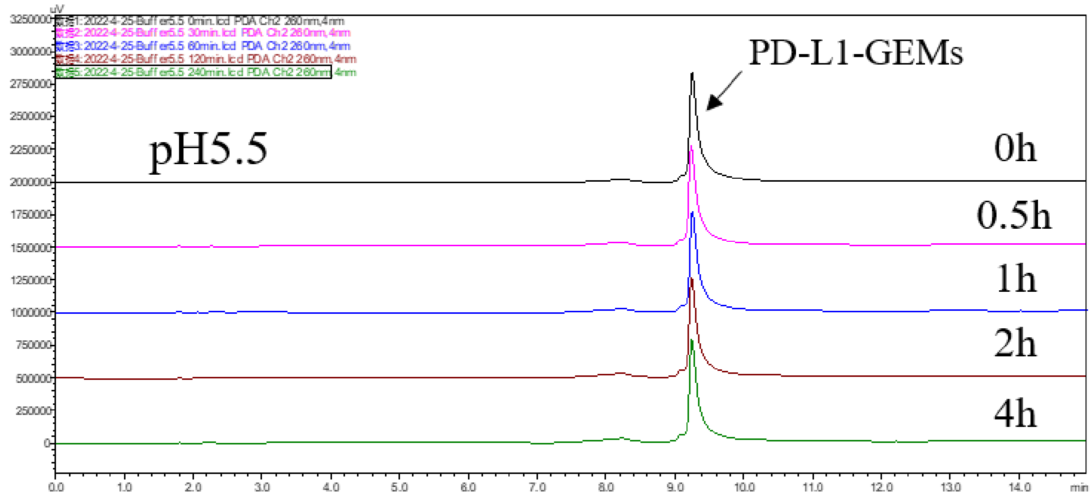

Figure S20. The release of GEM from PD-L1-GEMs in pH 5.5 buffer.

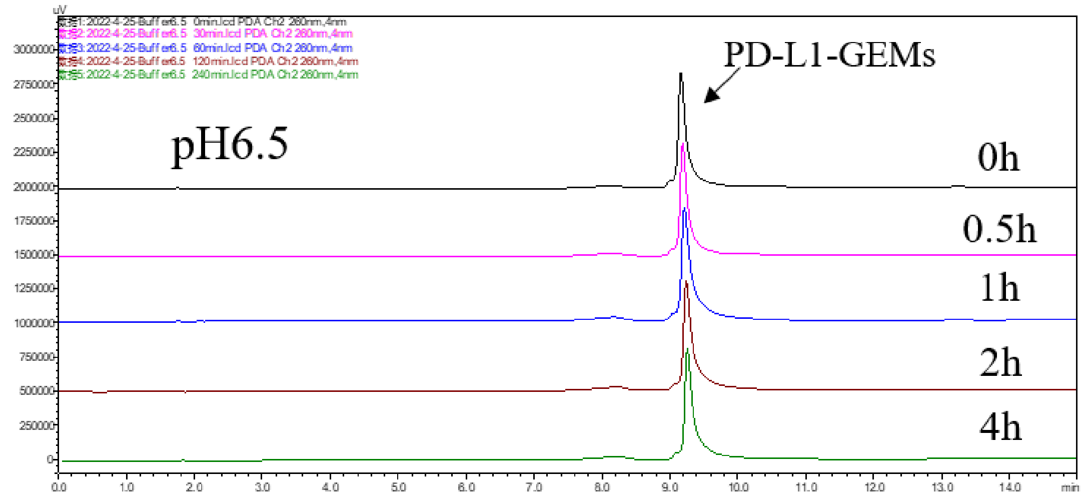

Figure S21. The release of GEM from PD-L1-GEMs in pH 6.5 buffer.

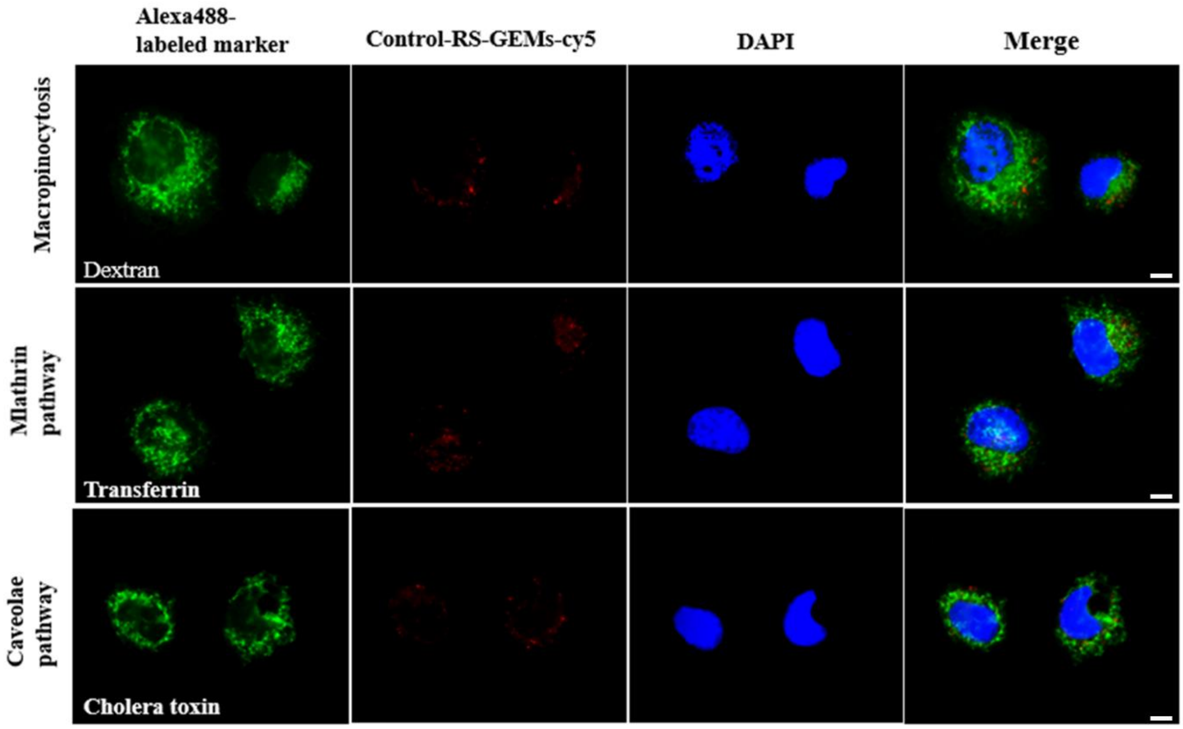

Figure S22. Internalization and trafficking of Control-RS-GEMs-cy5 in EJ cells. Photographs show Control-RS-GEMs-cy5 (red) co-localized with endocytic markers transferrin, cholera

toxin, and dextran labeled with Alexa Fluor 488 (green), respectively. The nuclei were counterstained with DAPI (blue). Scale bars: 10 μm.

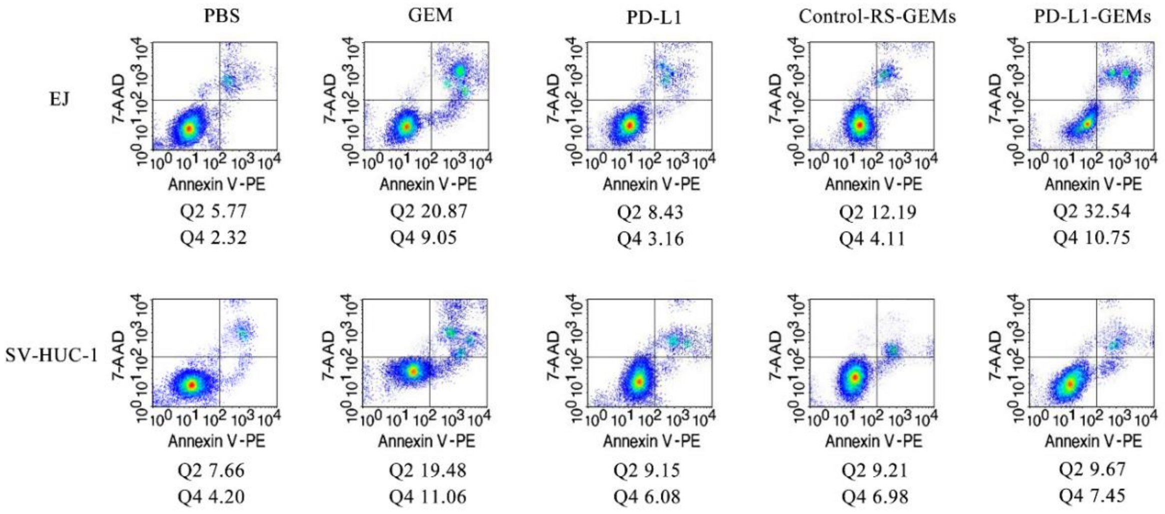

Figure S23. AnnexinV-PE+7-AAD double staining assay was used to detect the cytotoxicity of PD-L1-GEMs by flow cytometry.

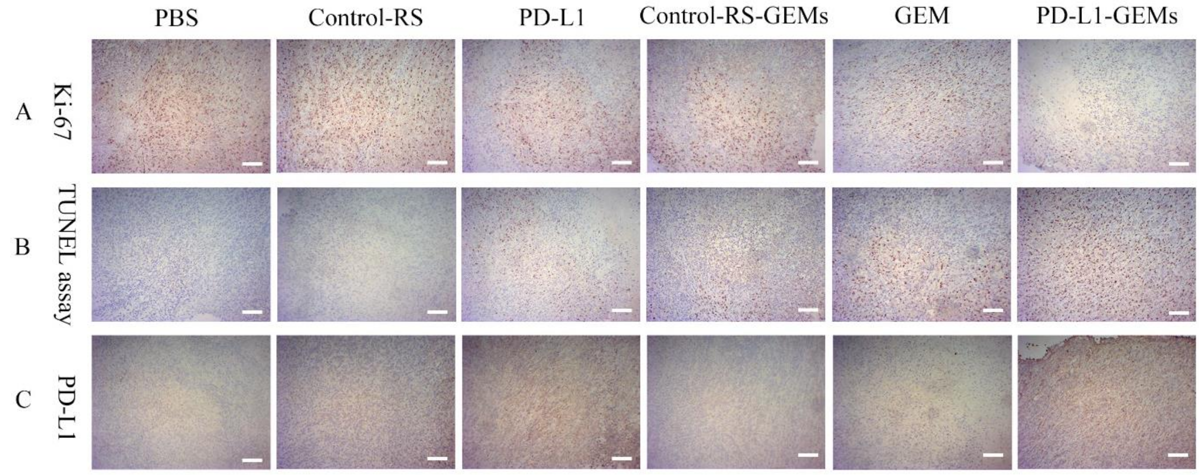

Figure S24. Biosafety assessment of PD-L1-GEMs in stained tumor sections. Tumor sections of different groups with (**A**) Ki67 staining (brown signal), (**B**) TUNEL staining (brown signal), (**C**) PD-L1 IHC staining. Scale bars: 100 μm.

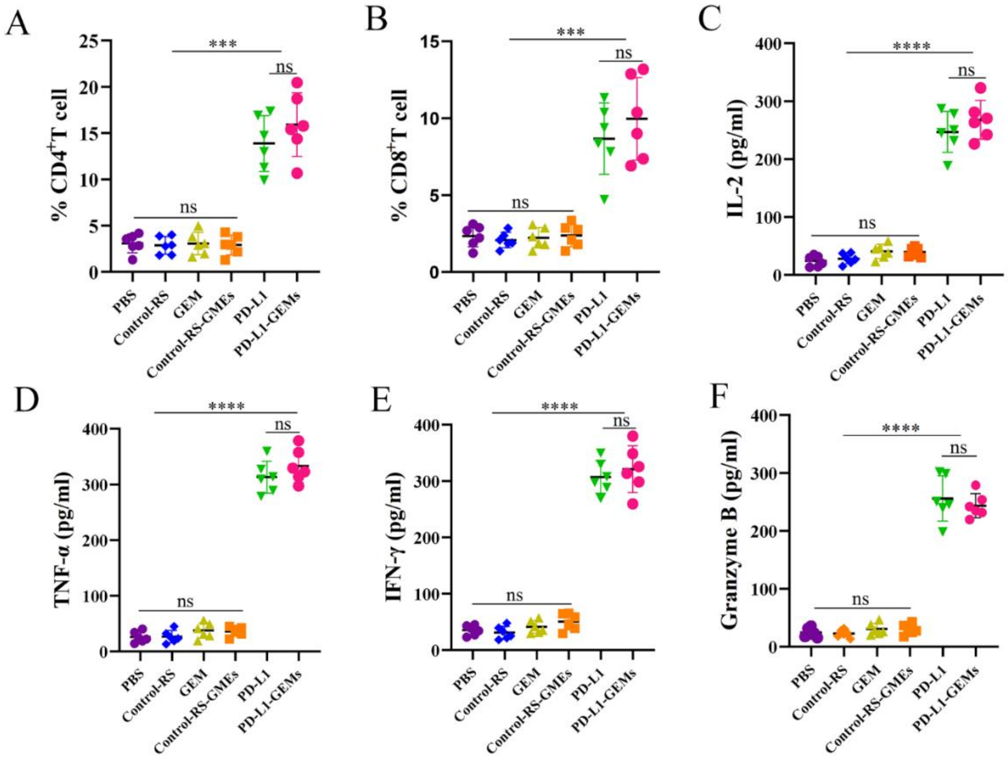

Figure S25. Lymphocytes infiltration and cytokines secretion in tumor tissues. (**A-B**) The infiltration of $CD4^+$ and $CD8^+$ T lymphocytes. (**C-F**) The secretion of IL-2, TNF-α, IFN-γ and Granzyme B. Data shown are the mean ± SD. (n = 6). ns: no significant difference. ***$p < 0.001$, ****$p < 0.0001$.

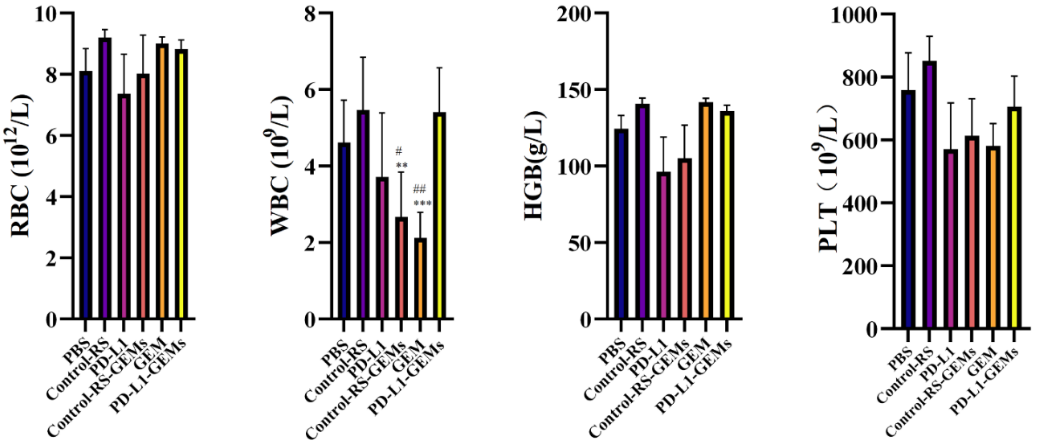

Figure S26. The biosafety assessment of PD-L1-GEMs in biochemical assays. RBCs: red blood cells, WBCs: white blood cells, HGB: hemoglobin, PLTs: platelets. Data are the mean ± SD, n =6. $^{\#}p < 0.05$ *vs*. the PBS group, $^{*}p < 0.05$ *vs*. the PD-L1-GEMs group; $^{\#\#}p < 0.01$ *vs* the PBS group, $^{**}p < 0.01$ *vs*. the PD-L1-GEMs group; $^{***}p < 0.001$ *vs*. the PD-L1-GEMs group.

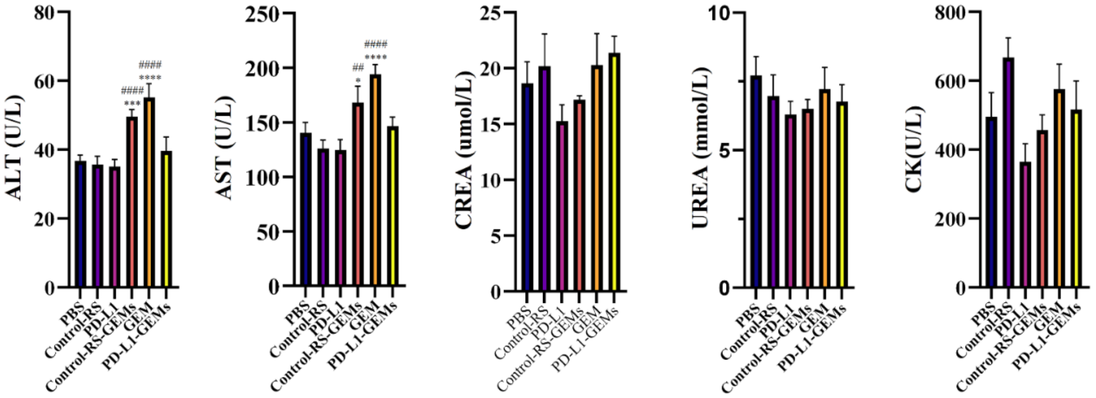

Figure S27. The biosafety assessment of PD-L1-GEMs in enzymes assays. ALT: alanine aminotransferase, AST: aspartate aminotransferase, CREA: creatinine, CK: creatine phosphokinase. Data are the mean ± SD, n = 6; $^{\#}p < 0.05$ *vs*. the PBS group, $^{*}p < 0.05$ *vs*. the

PD-L1-GEMs group; $^{\#\#}p < 0.01$ *vs*. the PBS group, $^{***}p < 0.001$ *vs*. the PD-L1-GEMs group; $^{\#\#\#\#}p < 0.0001$ *vs*. the PBS group, $^{****}p < 0.0001$ *vs*. the PD-L1-GEMs group.

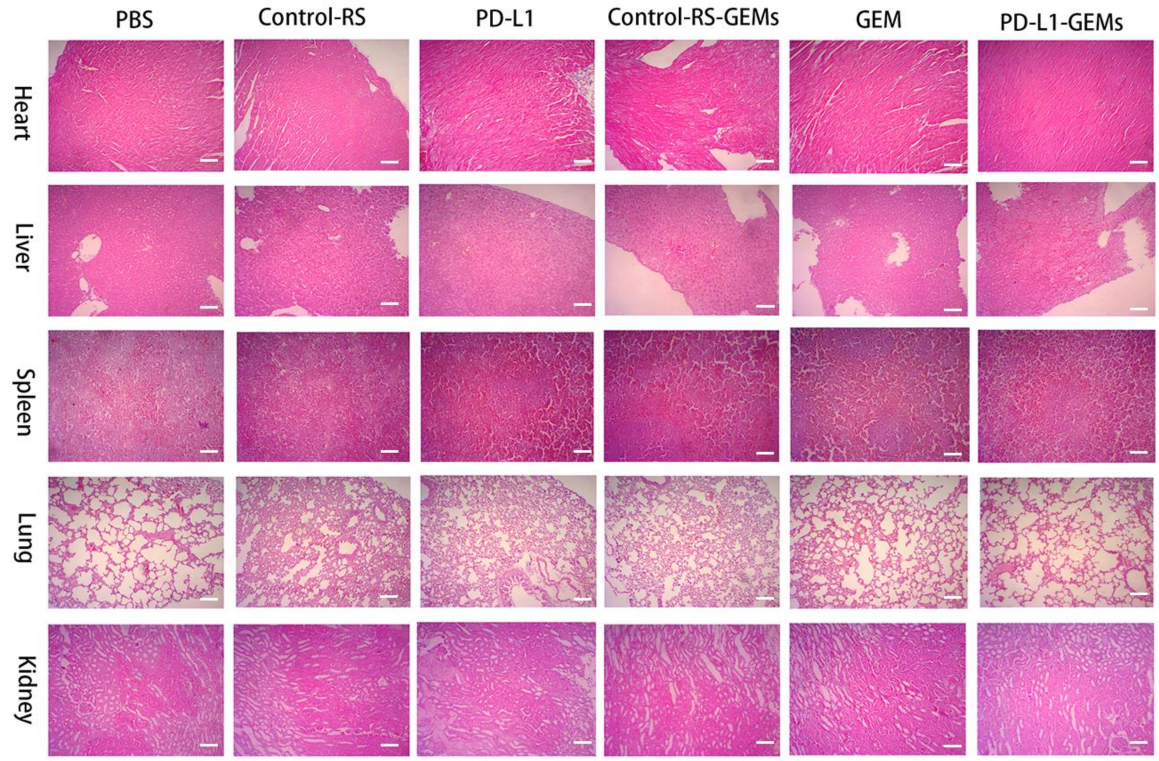

Figure S28. The biosafety assessment of PD-L1-GEMs in the staining of tissue sections. H&E staining analysis of heart, liver, spleen, lung and kidney tissues in the different groups. Scale bars: 100 μm.

Table S1. Relationship between PD-L1 expression and clinicopathologic characteristics in bladder cancer patients.

Characteristic	Number of patients (n = 148)	PD-L1 expression of bladder cancer cells: Negative (%) (n = 72)	Positive (%) (n = 76)	*P* value[a]
Age(years)				0.052
≤60[b]	62	36(58.1)	26(41.9)	
＞60	86	36(41.9)	50(58.1)	
Gender				0.084
Male	125	57(45.6)	68(54.4)	
Female	23	15(65.2)	8(34.8)	
Tumor size(cm)				0.964
≤3.8[c]	84	41(48.8)	43(51.2)	
＞3.8	64	31(48.4)	33(51.6)	
Tumor multiplicity				0.716
Unifocal	39	18(46.2)	21(53.8)	
Multifocal	109	54(49.5)	55(50.5)	
Tumor grade				**0.005**
Low	59	37(62.7)	22(37.3)	
High	89	35(39.3)	54(60.7)	
pT status				**＜0.001**
pT1	44	38(86.4)	6(13.6)	
pT2	52	23(44.2)	29(55.8)	
pT3/pT4	52	11(21.2)	41(78.8)	
pN status				**0.002**

pN-	123	67(54.5)	56(45.5)
pN+	25	5(20.0)	20(80.0)

[a]Chi-square test. [b]mean age. [c]mean size.

Table S2. Univariate analysis of PD-L1 and clinical features in bladder cancer patients.

Feature	All cases	HR (95% CI)	*P* value[a]
Age(years)			0.39
≤60[b]	62	1	
＞60	86	1.288 (0.723-2.296)	
Gender			0.934
Male	125	1	
Female	23	0.967 (0.435-2.150)	
Tumor size(cm)			0.596
≤3.8[c]	84	1	
＞3.8	64	1.166 (0.661-2.055)	
Tumor multiplicity			0.998
Unifocal	39	1	
Multifocal	109	1.001 (0.545-1.837)	

Tumor grade			**＜0.001**
Low	59	1	
High	89	4.092 (1.978-8.466)	
pT status			**0.013**
pT1	44	1	
pT2	52	2.029 (0.847-4.860)	
pT3/pT4	52	3.365 (1.454-7.789)	
pN status			**＜0.001**
pN-	123	1	
pN+	25	3.878 (2.110-7.127)	
PD-L1 expression			**＜0.001**
Negative	46	1	
Positive	102	3.983 (2.037-7.789)	

[a]Chi-square test. [b]mean age. [c]mean size. HR: hazard ratio. CI: confidence interval.

Table S3. Multivariate analysis of PD-L1 and clinical features in bladder cancer patients.

Feature	Hazards ratio	95% CI[a]	*P* value
Tumor grade (low *vs.* high)	2.982	1.464-6.074	**0.003**
pT status (pT1 *vs.* pT2 *vs.* pT3/pT4)	1.179	0.751-1.849	0.474
pN status (pN- *vs.* pN+)	1.933	0.950-3.932	**0.037**
PD-L1 expression (negative *vs.* positive)	3.122	1.450-6.725	**0.004**

[a]CI: confidence interval.

Table S4. Aptamer sequences used in the study.

**Name**	**Sequences and modification (5' to 3')**
PD-L1	TT TAC AGG TTC TGG GGG GTG GGT GGG GAA CCT GTT TT
Control-RS	TT ATT ACC TCT AAA TCA CTG CTC TGT AAC ATG GTC GCG CTA GG TT

PD-L1-FITC	TT TAC AGG TTC TGG GGG GTG GGT GGG GAA CCT GTT TT(FITC)
Control-RS-FITC	TT ATT ACC TCT AAA TCA CTG CTC TGT AAC ATG GTC GCG CTA GG TT(FITC)
PD-L1-cy5	TT TAC AGG TTC TGG GGG GTG GGT GGG GAA CCT GTT TT (cy5)
Control-RS-cy5	TT ATT ACC TCT AAA TCA CTG CTC TGT AAC ATG GTC GCG CTA GG TT (cy5)
PD-L1-GEMs	TT TAC AGG TTC TGG GGG GTG GGT GGG GAA CCT GTT MMM TT
Control-RS-GEMs	TT ATT ACC TCT AAA TCA CTG CTC TGT AAC ATG GTC GCG CTA GG MMM TT

Table S5. Tumor suppression effect of different treatment on bladder histopathologic changes in SD rat bladders of different groups.

Group	No.of rats sacrificed	Normal (T0)	Cancer in situ or Noninvasive papillary carcinoma (Tis/Ta)	Subepithelial connective tissue invasive bladder cancer (T1)	Muscle invasive bladder cancer (≥T2)
PBS control	10	0	1	1	8
PD-L1	10	0	2	3	5
Control-RS	10	0	1	2	7
GEM	10	1	4	2	3
Control-RS-GEMs	10	2	3	2	3
PD-L1-GEMs	10	5	3	2	0

Table S6. Hematological values and serum biochemical values of treated mice (Mean ± SD).

Group	PBS	Control-RS	PD-L1	Control-RS-GEMs	GEM	PD-L1-GEMs
RBC (10^9/L)	8.9 ± 0.5	9.4 ± 0.8	7.36 ± 2.8	8.1 ± 2.1	9.1 ± 0.46	9.1 ± 2.8
WBC (10^{12}/L)	5.7 ± 1.9	5.4 ± 1.3	3.7 ± 1.5	2.7 ± 1.1	1.95 ± 0.7	4.7 ± 1.1
PLTs (10^9/L)	874.4 ± 69.9	801.4 ± 165.7	781.2 ± 186	701.1 ± 217.0	644.4 ± 86.5	784.4 ± 154.5
HGB(g/L)	132.8 ± 5.1	140.6 ± 9.35	130.2 ± 20.3	139.8 ± 5.1	130.3 ± 86.5	139.3 ± 4.39
ALT (U/L)	37.1 ± 1.6	35.7 ± 2.3	35.1 ± 2.1	49.6 ± 2.0	55.02 ± 3.9	39.6 ± 4.1
AST (U/L)	140.5 ± 9.4	126.1 ± 7.5	124.5 ± 9.6	168.0 ± 15.1	194.5 ± 9.0	146.6 ± 8.1
CREA (umol/L)	18.6 ± 2.1	20.1 ± 2.8	15.0 ± 1.4	17.2 ± 0.4	15.0 ± 1.4	21.4 ± 1.6
UREA (mmol/L)	7.7 ± 0.6	6.6 ± 1.0	6.3 ± 0.4	6.5 ± 0.3	6.23 ± 0.4	6.7 ± 0.6
CK (U/L)	495.4 ± 156.9	667.9 ± 126.0	350.1 ± 11.9	457.0 ± 98.2	350.1 ± 116.2	516.4 ± 185.7

RBC: red blood cell, WBC: white blood cells, PLT: platelet, HGB: hemoglobin, ALT: alanine aminotransferase, AST: aspartate aminotransferase, CREA: creatinine, and CK: creatine phosphokinase.

References

1. Wang, R.; Zhu, G.; Mei, L.; Xie, Y.; Ma, H.; Ye, M.; Qing, F. L.; Tan, W., *J Am Chem Soc* **2014,** *136* (7), 2731-4. DOI 10.1021/ja4117395.

2. Xiang, W.; Peng, Y.; Zeng, H.; Yu, C.; Zhang, Q.; Liu, B.; Liu, J.; Hu, X.; Wei, W.; Deng, M.; Wang, N.; Liu, X.; Xie, J.; Hou, W.; Tang, J.; Long, Z.; Wang, L.; Liu, J., *Biomater Res* **2022,** *26* (1), 74. DOI 10.1186/s40824-022-00328-9.
